# Relationship of self-reported body size and shape with risk for prostate cancer: A UK case-control study

**DOI:** 10.1371/journal.pone.0238928

**Published:** 2020-09-17

**Authors:** Mohammad Aladwani, Artitaya Lophatananon, Fredie Robinson, Aneela Rahman, William Ollier, Zsofia Kote-Jarai, David Dearnaley, Govindasami Koveela, Nafisa Hussain, Reshma Rageevakumar, Diana Keating, Andrea Osborne, Tokhir Dadaev, Mark Brook, British Association of Urological Surgeons’ Section of Oncology, Rosalind Eeles, Kenneth R. Muir

**Affiliations:** 1 Division of Population Health, Health Services Research and Primary Care, School of Health Sciences, Faculty of Biology, Medicine and Health, The University of Manchester, Manchester, United Kingdom; 2 School of Medicine, University Malaysia Sabah, Sabah, Malaysia; 3 Shaheed Mohtarma Benazir Bhutto Medical University, Bakrani, Pakistan; 4 School of Healthcare Science, Faculty of Science and Engineering, Manchester Metropolitan University, Manchester, United Kingdom; 5 The Institute of Cancer Research, London, United Kingdom; 6 The British Association of Urological Surgeons Ltd, London, United Kingdom; 7 The Royal Marsden NHS Foundation Trust, Sutton, United Kingdom; University of Zurich, SWITZERLAND

## Abstract

**Introduction:**

Previous evidence has suggested a relationship between male self-reported body size and the risk of developing prostate cancer. In this UK-wide case-control study, we have explored the possible association of prostate cancer risk with male self-reported body size. We also investigated body shape as a surrogate marker for fat deposition around the body. As obesity and excessive adiposity have been linked with increased risk for developing a number of different cancers, further investigation of self-reported body size and shape and their potential relationship with prostate cancer was considered to be appropriate.

**Objective:**

The study objective was to investigate whether underlying associations exist between prostate cancer risk and male self-reported body size and shape.

**Methods:**

Data were collected from a large case-control study of men (1928 cases and 2043 controls) using self-administered questionnaires. Data from self-reported pictograms of perceived body size relating to three decades of life (20’s, 30’s and 40’s) were recorded and analysed, including the pattern of change. The associations of self-identified body shape with prostate cancer risk were also explored.

**Results:**

Self-reported body size for men in their 20’s, 30’s and 40’s did not appear to be associated with prostate cancer risk. More than half of the subjects reported an increase in self-reported body size throughout these three decades of life. Furthermore, no association was observed between self-reported body size changes and prostate cancer risk. Using ‘symmetrical’ body shape as a reference group, subjects with an ‘apple’ shape showed a significant 27% reduction in risk (Odds ratio = 0.73, 95% C.I. 0.57–0.92).

**Conclusions:**

Change in self-reported body size throughout early to mid-adulthood in males is not a significant risk factor for the development of prostate cancer. Body shape indicative of body fat distribution suggested that an ‘apple’ body shape was protective and inversely associated with prostate cancer risk when compared with ‘symmetrical’ shape. Further studies which investigate prostate cancer risk and possible relationships with genetic factors known to influence body shape may shed further light on any underlying associations.

## Introduction

Prostate cancer is the most prevalent cancer in men [1]. It is also the third most common cancer-specific cause of death for men living in Europe [2, 3]. In 2016, it accounted for approximately one quarter of all cancers diagnosed in men within the UK [4]. Apart from the established cancer risk factors, such as age, ethnicity and family history of prostate cancer in first degree-relatives, other potential risk factors include height, obesity/high body mass index (BMI) and levels of insulin-like growth factor-I [5–7].

Over the last few decades, obesity has increased by approximately 30% in European men [8, 9]. This has been linked to increased risk for developing several chronic diseases and cancers [10]. Extensive studies have investigated the association of both obesity and body size with prostate cancer risk. However, this relationship remains inconclusive [11–15]. Anthropometrics that have been used to measure obesity and body adiposity include waist circumference, waist-hip ratio and BMI [16]. The majority of epidemiologic studies investigating prostate cancer risk have used BMI to evaluate obesity rather than body fat distribution [3]. Previous studies have suggested that high BMI is associated with increased risks for advanced, aggressive and fatal prostate cancer [13, 15, 17–22]. In contrast, other studies have observed a decreased risk of localised/indolent cancer [13, 15, 23–25]. A large meta-analysis consisting of 27 prospective studies of prostate cancer observed no or weak association between BMI and total prostate cancer [26]. Similar findings have come from another systematic review examining the exposure in early adult life [27]. These conflicting results may in part be due to the fact that BMI has been criticised for its inaccuracy in measuring obesity and its ability to differentiate adipose and non-adipose tissues [28, 29]. This suggests that any association could be dependent on particular disease subtypes and the age of exposure [6, 12, 13, 30].

Both body shape and body size have often been used to describe the characteristics of the human body in health-related research. Defining obesity or adiposity through the use of clinical judgement including a consideration of body size appearance provides an alternative approach for determining the wider distribution of fat tissue over time.

The issue of whether weight change during adulthood is more strongly associated with prostate cancer than cross-sectional ‘current’ adiposity has not as yet been fully explored [31, 32]. Prostate cancer is characterised as being a slow developing disease. Thus the age that obesity develops in early adult life may be an important factor within the aetiology of this cancer [27, 33–35]. Moreover, early changes in prostate tissue have been seen in men during their early adulthood, suggesting that body size over lifetime is important [33, 36]. Adult weight change is a dynamic measure that could reflect imbalances in weight over time and it is thought to be more accurate than a static measure of adiposity such as BMI [19, 37]. However, these studies have reported inconsistent results [19, 31, 32]. Some studies found positive associations between weight gain and prostate cancer [38] whereas others have found an inverse association [39] or no association at all [14, 21]. In this study we specifically address the issues of whether male self-reported body size and overall body shape and self-reported body size and its change across three decades of life are associated with prostate cancer risk.

## Methods

The ‘Prostate Cancer Study on Gene-Environment Interactions’ is a large scale case-control study identifying and investigating potential risk factors for the development of prostate cancer in the UK. The study used a self-administered questionnaire and written informed consent was obtained from each participant. Cases comprised adult men >36 years at diagnosis with histologically confirmed prostate cancer. Male adult controls were selected from the same general practices as cases. Eligible controls were men without history of prostate cancer and were within an age range of ±5 years of cases. This study received ethical committee approval MREC/99/4/013 (Trent Research Ethics Committee), 07/MRE04/29 (Nottinghamshire County Teaching and Primary Care Trust).

Epidemiological data were collected for two time periods; the first between 1997–2004 and the second between 2007–2009. In the second period, some additional questions were added and other questions expanded within the questionnaire to provide more in-depth information, including information on body shape. This was done following a preliminary analysis of data collected from the first period. Data collection from the two time periods involved different subjects and no repeated measurements were performed. Individuals did not contribute their data more than once.

**Fig 1:**
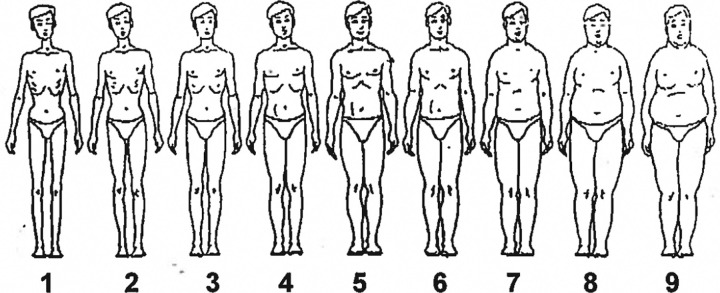
Pictogram with silhouette drawings used for recalling self-reported body size at each decade 20s, 30s, and 40s (taken from Stunkard et al, 1983).

Data on education was based on the UK educational system and social class was based on the UK occupational social class classification. Data on self-reported body size were available from both periods, but data on body shape were only available from the second period of data collection. Self-reported body size at different ages was assessed using a pictogram (Fig 1) with drawings of body silhouettes of nine different sizes ranging from 1 (very thin) to 9 (severely obese) [40]. Subjects were asked to recall information relating to their self-reported body size during their 20’s, 30’s and 40’s. Cases and controls were asked to rate their perceived body size for the last 5 year period prior to diagnosis in case group and for the last 5 years prior to receiving the questionnaire in control group. Participants were excluded from the analysis if there were incomplete data (i.e. missing data for any decade). This was done to ensure each participant has data to investigate self-reported body size changes throughout decades. 1928 cases and 2043 controls were available for the analysis of self-reported body size in the 20’s and 30’s. Six subjects were younger than 40 years of age at the time data were collected; hence the number of cases and controls eligible for self-reported body size analysis in the 40’s were 1924 and 2041 respectively. Ordinal scale data (scale of 1 to 9) for self-reported body size at age 20’s, 30’s, 40’s were grouped into ‘thin’ (scale 1–3), ‘medium’ (scale 4–6) and ‘large’ (scale 7–9).

To explore the effect of self-reported body size increase during adulthood on prostate cancer risk, we restricted our analysis to include only subjects whose self-reported body size remained as medium size from 20’s to 40’s as our reference group and subjects whose self-reported body size was medium both in their 20s and 30s but increased to large in their 40’s as our exposed group (Fig 1). There are 1057 cases and 1099 controls.

For body shape, participants were asked to select their body shape in four different forms (apple, pear, oval and symmetrical) that best represented their body shape throughout their life. Description of each body shape type was provided to aid subject’s understanding on its meaning (‘Apple’ shape is where body fat is distributed mainly around the central abdominal area; ‘Pear’ shape is where body fat is distributed mainly around the hip and thigh; ‘Oval’ shape is where body fat is distributed around the neck, chest, abdominal area and thigh; ‘Symmetrical’ shape is where the person has lean body with no fat). The numbers of subjects included in this particular analysis were 1329 cases and 812 controls.

### Statistical analysis

Logistic regression analysis was performed on the data using Stata version 15.0 [41]. Odds ratios (ORs) and 95% confidence intervals (CIs) were estimated for total prostate cancer risk. Forward stepwise logistic regression was performed on demographic factors to identify potential confounders. The final multivariate logistic regression model included education, ethnicity, study phase (I and II) and family history of prostate cancer in first-degree relatives. Multivariable logistic regression was fitted with all confounders. Age was also included as an *a-priori* variable in all regression models. For self-reported body size, medium size was used as reference category and for pattern of change, no change from 20s to 40s was used as reference group. In the multivariate model, self-reported body size at age 30’s and 40’s were adjusted further to self-reported body size at age 20’s to minimise the effect of correlation between self-reported body size at age 20’s to age 30’s and 40’s. For body shape, symmetrical shape was used as reference category. Estimated risks were obtained from multivariate logistic regression models. A significant odds ratio is considered when 95% C.I does not include 1.

### Sensitivity analysis

We collected data on current BMI from both periods. A sensitivity analysis was performed to explore if self-reported body size can be used as a proxy marker for BMI. We used the self-reported body size and BMI reported during the last 5 years prior to completing the questionnaire only in the controls due to the fact that prostate cancer may have affected current BMI in cases. Data were available in 766 controls. BMI as a continuous variable was normally distributed hence we applied Analysis of Variance (ANOVA) to explore the differences among group means. A finding was deemed to be statistically significant when the P-value was less than 0.05.

### Study power

As they are no previous studies on body shape and prostate cancer, we computed our study power based on exposure in our study. Our study of 1329 case and 812 controls with a probability of exposure (apple body shape) among controls of 0.62, had a 95% study power to detect odds ratios for disease of 0.72 or 1.41 [42].

## Results

The overall study response rates after initial consent to complete the questionnaire were 85.0% for cases and 74.4% for controls. Table 1 shows the study population characteristics. The median age for both case and control subjects was 60 and 59 years respectively. The vast majority of study subjects described themselves as white (98%).

**Table 1:** Demographic and social characteristics of participants in the prostate cancer study on gene-environment interactions.

Characteristics	Cases (n = 1,928)	Controls (n = 2,043)	OR of prostate cancer	(95% CI)
	Median	Median		
**Age (years)**	60 (range 36–84)	59 (range 36–76)		
	**n (%)**	**n (%)**		
**Marital Status**				
Married or partnership	1,585 (82.2%)	1,691 (82.8%)	-Ref-	
Divorced, separated or widowed	227 (11.8%)	260 (12.7%)	0.93	0.77–1.13
Single	89 (4.6%)	68 (3.3%)	1.39	1.01–1.93
Missing	27 (1.4%)	24 (1.2%)		
**Education**				
No qualifications	433 (22.5%)	558 (27.31%)	-Ref-	
GCSE, O levels or equivalent	357(18.5%)	342 (16.74%)	1.35	1.11–1.64
A levels, higher or equivalent	132 (7.0%)	148 (7.24%)	1.16	0.89–1.51
Higher or professional qualification e.g. degree, HND	716 (37.0%)	742 (36.32%)	1.25	1.06–1.47
Others	252 (13.0%)	229 (11.21%)	1.42	1.14–1.76
Missing	38 (2.0%)	24 (1.17%)		
**Ethnicity**				
White	1,832 (95.0%)	2,000 (97.9%)	-Ref-	
Black	29 (1.5%)	4 (0.2%)	8.1	2.84–23.12
Asian	13 (0.7%)	7 (0.34%)	1.99	0.79–5.02
Other	26 (1.4%)	13 (0.64%)	2.19	1.12–4.29
Missing	28 (1.4%)	19 (0.93%)		
**Social class**				
I	236 (12.2%)	224 (11%)	-Ref-	
II	797 (41.3%)	851 (41.7%)	0.89	0.72–1.10
IIIN	193 (10.0%)	208 (10.2%)	0.88	0.67–1.15
IIIM	499 (26.0%)	528 (25.8%)	0.90	0.73–1.13
IV	108 (5.6%)	111 (5.4%)	0.93	0.67–1.28
V	18 (0.9%)	31 (1.5%)	0.56	0.30–1.02
Missing	77 (4.0%)	90 (4.4%)		
**Family history of prostate cancer**				
No	1,312 (68.0%)	1,880 (92.0%)	-Ref-	
Yes	533 (27.7%)	100 (4.9%)	7.61	6.08–9.54
Missing	83 (4.3%)	63 (3.1%)		

*Unadjusted OR. The rest of ORs were adjusted for age.

Table 2 summarises the number of subjects and their self-reported body size at each of the three decades of their life. The majority of participants was medium across all three decades in both case and control groups.

**Table 2:** Self-reported body sizes at each decade among cases and controls.

**Body size at 20’s**	**Cases**	**Controls**	**OR of prostate cancer**^a^	**OR of prostate cancer**^b^
Medium	1,159 (60.1%)	1,208 (59.1%)	-Ref-	-Ref-
Thin	690 (35.8%)	736 (36.0%)	0.97 (0.85–1.11)	1.10 (0.95–1.28)
Large	79 (4.1%)	99 (4.9%)	0.84 (0.62–1.14)	0.95 (0.66–1.35)
Body size at 30’s *				
Medium	1,497 (77.7%)	1,573 (77.0%)	-Ref-	-Ref-
Thin	255 (13.2%)	273 (13.4%)	0.97 (0.80–1.17)	0.97 (0.77–1.22)
Large	176 (9.1%)	197 (9.6%)	0.96 (0.77–1.19)	1.00 (0.77–1.30)
Body size at 40’s *				
Medium	1,291 (67.1%)	1310 (64.2%)	-Ref-	-Ref-
Thin	70 (3.6%)	91 (4.5%)	0.77 (0.56–1.06)	0.85 (0.58–1.23)
Large	563 (29.3%)	640 (31.4%)	0.91 (0.80–1.05)	1.00 (0.85–1.75)

^a^ Age-adjusted regression model

^b^ Multivariate adjusted regression model for age, education, ethnicity, study phase and family history of prostate cancer

*Body size at 30’s and 40’s adjusted further to body size at 20’s in the multivariate model

Table 3 summarises odds ratios of self-reported body size changes and prostate cancer risk. Both cases and controls have similar percentage of self-reported body size change from medium to large in their 40’s (~30%). The result suggests that there is no association with cancer risk for subjects whose self-reported body size increased from medium to large as compared to subjects with medium self-reported body size throughout their adulthood.

**Table 3:** Estimated risk of self-reported body size changes and prostate cancer risk.

Group	Cases	Controls	OR of prostate cancer^a^ (95%CI)	OR of prostate cancer^b^ (95%CI)
**Body size remains thin or medium throughout adulthood**	738	758	-Ref-	-Ref-
**Body size increase to large in their 40s**	319	341	0.97 (0.81–1.17)	1.07 (0.87–1.33)
**Total**	1,057	1,099		

^a^ Age-adjusted regression model

^b^ Multivariate adjusted regression model for age, education, ethnicity, study phase and family history of prostate cancer

Table 4 presents estimated risks of different self-reported body shape and prostate cancer risk. Compared to symmetrical shape, subjects with an apple shape were at 27% risk reduction (OR in the fully adjusted model = 0.73 with 95% CI 0.57–0.92). Both pear and oval shape did not show any association with prostate cancer risk in the fully adjusted model of 1.44 (95% CI 0.77–2.69) and 0.82 (95% CI 0.59–1.13) respectively. Although, the association is not significant, but the direction of effect suggested that adipose tissue distributed around the hip and thigh (pear) is at higher risk, while abdominal fat distribution (apple, and oval) is at lower risk.

**Table 4:** Odd ratios of self-reported body shape on prostate cancer risk.

Self-reported body shape	Case	Control	OR of prostate cancer^a^ (95%CI)	OR of prostate cancer^b^ (95%CI)
**Symmetric**	349	173	-Ref-	-Ref-
**Apple**	735	504	0.67 (0.53–0.83)	0.73 (0.57–0.93)
**Pear**	51	17	1.57 (0.87–2.85)	1.47 (0.78–2.76)
**Oval**	194	118	0.76 (0.56–1.02)	0.82 (0.59–1.14)

^a^ Age-adjusted regression model

^b^ Multivariate adjusted regression model for age, education, ethnicity, and family history of prostate cancer

Results from sensitivity analysis (only in the control group) using ANOVA test is presented in Table 5. The significant p-value suggested that mean BMI in each group is a statistically significant difference. BMI increases with increased self-reported body size indicative of a good proxy between BMI and body size.

**Table 5:** BMI and self-reported body size in control group*.

Body size	Number	Mean	Std. Dev.
**2**	6	20.23	1.69
**3**	17	21.78	2.07
**4**	48	22.97	1.67
**5**	103	24.02	2.39
**6**	168	25.44	2.07
**7**	254	27.46	3.13
**8**	135	30.18	3.56
**9**	35	34.14	4.49

*ANOVA F-test P-value <0.05

## Discussion

Three key areas potentially relating to increased risk for prostate cancer were explored in this study; self-reported body size at early and mid-adulthood, self-reported body size changes over decades in life, and self-identified body shape.

Self-reported body size (thin, medium, and large) ranging across three decades (20’s, 30’s and 40’s) was explored and analysis suggested no associations between the self-reported body size at each stage of life among cases and control group and risk of prostate cancer however our analysis could be underpowered given the relatively small numbers in the 20’s/large and 40’s/thin category. Furthermore, the analysis suggested that 55% of both case and control subjects had a history of changes in self-reported body size. Our *ad hoc* analysis also showed that the magnitude of changes of self-reported body size from age 20’s to 40’s varies between individuals (result not shown here). Approximately 53% of those self-reported body size changes were of increase in size (either for both periods-20s to 30s and 30s to 40s or at 20s to 30s and no change in 30s to 40s). The possible explanation for increase in body size is because of decreased metabolic rate with ageing and accumulation over the years of unburned calorie intakes. Environmental factors such as eating high-fat foods or lack of exercise, as well as Sedentary Lifestyle Syndrome (SeDS) could also be accountable for increasing in body size [43]. These possible explanations are compatible with the considerable social and life style changes that have occurred across the UK over the last 30 years.

The findings of previous studies regarding obesity at early and mid-adulthood are inconclusive. Our results are consistent with the majority of epidemiologic studies that found no associations between self-reported body size in early as well as middle to late adulthood and prostate cancer risk [14, 21, 27, 39, 44, 45]. More recently, a research group (the Prostate Cancer Association Group to Investigate Cancer Associated Alterations in the Genome (PRACTICAL) consortium) investigated potential causal relationship between BMI and prostate cancer using genetic approaches to analyse 20848 cases and 20214 controls. This also failed to identify any significant associations between BMI and prostate cancer [46]. Our study also did not find any association between changes in self-reported body size over decades (increase in self-reported body size to large in the 40’s compared to remains medium throughout) and prostate cancer risk. This finding is inconsistent with several other studies where some relationships with prostate cancer were observed [14, 19, 24, 38, 47, 48]. This inconsistency could be due to the different measurements used by these studies which used actual weight, BMI or waist circumference to indicate the change in body size. In contrast, in our study we used pictograms as a surrogate for body size. We also performed analyses of BMI and perceived body size within different social class and education in the control group and the results suggested a very similar correlation to that seen in the main sensitivity analysis. Furthermore, the other studies used multiple parameters to measure body size when investigating the relationship of change of body size with prostate cancer. As such there was therefore a higher possibility of obtaining statistical significant findings in at least one of the measurement parameters. The other limitation is that our data is only limited to middle age (40s) hence this may not be the period in life that obesity associates with prostate cancer. Our results which failed to show association are in keeping with the majority of other studies that investigated the association between weight change and prostate cancer risk [12, 31, 32, 39, 44, 45, 49–53].

A limitation of using pictorial illustration is its inability to make an actual measurement of changes in body size in comparison with using other parameters such as weight, waist circumference or waist-hip ratio, BMI or body fat mass. As such, pictorial assessment of self-reported body size is relative, but it may be better for showing body size change over long time windows. Pictograms are considered to be a valid and useful method to assess self-reported body size and differentiate thin and obese individuals [54]. The Stunkard Figure Rating (SFR) scale of body size [40] tool has been validated for historic recall of body size and was used in a large European population to explore correlation between self-reported body silhouettes and the previously measured (9–23 years) BMI [55]. The authors reported an area under the curve of 0.92 (95% CI 0.87, 0.97) in women and 0.85 (95% CI 0.75, 0.95) in men for identifying obesity at age 30 using body silhouettes vs previously measured BMI at age 30 (±2y). The findings were also similar for previously self-reported BMI, 0.92 (95% CI 0.88, 0.95) and 0.90 (95% CI 0.85, 0.96) in women and men respectively. Another study assessing adolescent body size found that Stunkard’s method was a useful indicator in absence of measured BMI [56]. It is also has been reported that recalled body size using pictograms showed a strong correlation with measured weight at age 20–40 years with a correlation ranging from 0.51 to 0.95 [57–59]. Our result from sensitivity analysis in controls suggested that pictogram can potentially be used for recall of body size. Nevertheless, personal perception of body size of each individual could introduce bias such as classification bias.

Cohort studies often obtain more valuable data by longitudinally measuring and recording body weight, waist/hip circumference and body fat mass. Implementing this approach was not possible in our study. Some medical conditions, such as hypo or hyperthyroidism, can affect body size. However the prevalence of both these conditions in the UK is low (1–2% for both conditions) [60] and therefore unlikely to affect our results. As our study is subject to classification bias, we opted to broadly group body size into three groups to minimise any bias; i.e. thin, medium and large.

We are not aware of any published research on the prevalence of different types of body fat distribution in the population. However waist and chest circumference measurement in males are the closest for describing whether a person shape can be described as ‘apple’ or be a proxy of central adiposity [61]. Male shape seems to remain highly stable throughout adult life, therefore it is reasonable to assume that characteristic of body fat distribution also remains the same.

Our results suggest that subjects with an ‘apple’ shape indicative of body fat distributed mainly around the abdomen, were at reduced risk with both adjusted and unadjusted when compared to those with a ‘symmetrical’ shape. However, the ‘pear’ and ‘oval’ body shapes did not show any statistically significant associations. A recent cohort study reported by Barberio involving 26607 subjects, found central body adiposity to be more associated with cancer risk than overall body size [62]. Although the cohort examined the association with cancer in general, our results of self-identify body shape indicative of the distribution of fat tissue around the body suggested similar findings.

In contrast to ‘apple’ or ‘pear’ body shape, hip circumference indicates increased amounts of subcutaneous fat. Thus ‘apple’ body shape in actual measurement would predict wider waist circumference (WC) or higher waist to hip ratio (WHR). Studies using actual measurement have shown increased risk in advanced or high-grade prostate cancer in such individuals [3, 16, 30, 63, 64].

Several possible explanations have been proposed regarding association between central adiposity and prostate cancer. Adiposity can potentially impact through multiple hormonal pathways. Adiposity has been associated with higher levels of insulin, insulin like growth factor I, leptin, and inflammatory cytokines. It has also been linked with lower levels of adiponectin and free testosterone. All of these may impact on prostate cancer development and progression [20, 65–72]. Moreover, some studies showed that adiposity lowered the risk of non-aggressive prostate cancer while at the same time increased the risk for aggressive and high-grade prostate cancer [3, 5, 8, 14, 16, 20, 21, 24, 25, 30, 33, 73]. However other studies have observed weak or no association with prostate cancer and disease subtypes [12, 74–76].

As yet no other study reported in the literature has used body shape as proxy measure of body fat distribution to investigate possible associations with prostate cancer. Our findings suggest that abdominal fat deposition (apple body shape) maybe protective of prostate cancer.

Diabetes is known to be linked with obesity and also shows an inverse association with the risk of prostate cancer [77–79]. One of the limitations is that we collected data on diabetes only in period 2 with no details of diabetes type. However, we carried out logistic regression analysis incorporating diabetes in our model, our results remain the same. Likewise, we also investigated the association of both smoking and physical activity with prostate cancer and there were no associations. Therefore, we did not include these variables in our final model.

In this study, we used self-reported descriptions within the questionnaire to capture the types of body fat distribution. This approach is likely to be less accurate than using 3-dimensional body shape scanning as used in UK National Sizing survey [61] conducted in 2001 to 2002. This cross-sectional study of 9617 adults found that male body shape remained highly stable throughout adulthood. Such quantitative approaches may reveal further insights into the role and influence of lipidosity and its site of deposition on prostate cancer risk and development.

## Supporting information

S1 File Collaborators. (PDF)

S2 File Q_section 10. (DOCX)
