# Supplementary material for: Relationship of self-reported body size and shape with risk for prostate cancer: A UK case-control study
Source: PLoS One. 2020 Sep 17;15(9):e0238928. doi: 10.1371/journal.pone.0238928 (PMC7498010; doi:10.1371/journal.pone.0238928)
Supplement: S1 File — (PDF) [file pone.0238928.s001.pdf]

| Consultant | Surname | Hospital | Address | Postcode |
|---|---|---|---|---|
| Mr Z Abbasi | Abbasi | ROTHERHAM GENERAL HOSPITAL | Moorgate Road, Rotherham | S60 2UD |
| Mr Abdallah | Abdallah | WREXHAM MAELOR HOSPITAL | Croesnewydd Rd, Wrexham | LL13 7TD |
| Mr M Akhlil Abdul-Hamid | Abdul-Hamid | NOTTINGHAM CITY HOSPITAL | Hucknall Road, Nottingham | NG5 1PB |
| Mr S Abedin | Abedin | RUSSELLS HALL HOSPITAL | Pensnett Road, Dudley, West Midlands | DY1 2HQ |
| Mr Paul D Abel | Abel | CHARING CROSS HOSPITAL | Fulham Palace Road, London | W6 8RF |
| Professor Paul H Abrams | Abrams | SOUTHMEAD HOSPITAL | Westbury-on-Trym, Bristol, Somerset | BS10 5NB |
| Dr Fawzi A Adab | Adab | UNIVERSITY HOSPITAL OF NORTH STAFFORDSHIRE | The Cancer Centre, City General Site, Newcastle Road, Stoke-on-Trent | ST4 6QG |
| Mr Andrew Adamson | Adamson | ROYAL HAMPSHIRE HOSPITAL | Romsey Road, Winchester, Hants | SO22 5DG |
| Mr A Adeyoju | Adeyoju | STEPPING HILL HOSPITAL | Poplar Grove, Hazel Grove, Stockport | SK2 7JE |
| Mr Naveed Afzal | Afzal | DORSET COUNTY HOSPITAL | Williams Avenue, Dorchester | DT1 2JY |
| Mr Ernest K N Ahiaku | Ahiaku | YSBYTY GWYNEDD | Penrhosgarnedd, Bangor | LL57 2PW |
| Mr Munir Ahmed | Ahmed | PRINCESS ROYAL UNIVERSITY HOSPITAL | Farnborough Common, Orpington, Kent | BR6 8ND |
| Mr Mehmood Akhtar | Akhtar | HEREFORD COUNTY HOSPITAL | Stonebow Road , Hereford, Herefordshire | HR1 2BN |
| Mr Mohammed L Al Sudani | Al Sudani | KETTERING GENERAL HOSPITAL | Rothwell Road, Kettering, Northants | NN16 8UZ |
| Mr Ashar Alam | Alam | LUTON & DUNSTABLE HOSPITAL | Lewsey Road, Luton | LU4 0DZ |
| Dr Christopher Alcock | Alcock | STOKE MANDEVILLE HOSPITAL | Mandeville Road, Aylesbury, Buckinghamshire | HP21 8AL |
| Mr A Alexandrou | Alexandrou | YSBYTY GLAN CLWYD | Rhuddlan Rd, Bodelwyddan, Rhyl | LL18 5UJ |
| Dr Abdulla Alhasso | Alhasso | NEW VICTORIA HOSPITAL | 184 Coombe Lane West, Kingston upton Thames | KT2 7EG |
| Dr Zulfiqar Ali | Ali | GLAN CLWYD HOSPITAL | Bodelwyddan, Rhyl, Wales | LL18 5UJ |
| Mr David J Almond | Almond | CASTLE HILL HOSPITAL | Castle Road, Cottingham, East Yorkshire | HU16 5JQ |
| Dr Roberto Alonzi | Alonzi | MOUNT VERNON CENTRE FOR CANCER TREATMENT | Rickmansworth Road, Northwood, Middlesex | HA6 2RN |
| Dr Amir S M Al-Samarraie | Al-Samarraie | GLAN CLWYD HOSPITAL | Bodelwyddan, Rhyl, Wales | LL18 5UJ |
| Dr Al-Samerraie | Al-Samerraie | GLAN CLWYD HOSPITAL | Bodelwyddan, Rhyl, Wales | LL18 5UJ |
| Mr Waleed Al-Singary | Al-Singary | WORTHING HOSPITAL | Lyndhurst Road, Worthing, West Sussex | BN11 2DH |
| Mr Al-Sudani | Al-Sudani | KETTERING GENERAL HOSPITAL | Rothwell Road, Kettering, Northants | NN16 8UZ |
| Mr Pallavoor Anandaram | Anandaram | WREXHAM MAELOR HOSPITAL | Croesnewydd Road, Wrexham | LL13 7TD |
| Mr John Anderson | Anderson | ROYAL HALLAMSHIRE HOSPITAL | Glossop Road, Sheffield | S10 2JF |
| Mr Steven Andrews | Andrews | DORSET COUNTY HOSPITAL | Williams Avenue, Dorchester | DT1 2JY |
| Mr Henry Andrews | Andrews | MILTON KEYNES GENERAL HOSPITAL | Standing Way, Eaglestone, Milton Keynes | MK6 5LD |
| Mr Mike Aniah | Aniah | BRONGLAIS GENERAL HOSPITAL | Caradog Road, Aberystwyth | SY23 1ER |
| Mr Iqbal Anjum | Anjum | MILTON KEYNES GENERAL HOSPITAL | Standing Way, Eaglestone, Milton Keynes | MK6 5LD |
| Dr J Ansari | Ansari | HEARTLANDS HOSPITAL | Bordesley Green E, Birmingham | B9 5SS |
| Mr Ken Anson | Anson | ST GEORGE'S HOSPITAL | Blackshaw Road, Tooting, London | SW17 0RE |
| Dr Nicola A Anyamene | Anyamene | MOUNT VERNON CENTRE FOR CANCER TREATMENT | Rickmansworth Road, Northwood, Middlesex | HA6 2RN |
| Mr Ike Apakama | Apakama | GEORGE ELLIOTT HOSPITAL | College Street, Nuneaton, Warks | CV10 7BL |
| Dr F Aparcia | Aparcia | ST JAMES'S UNIVERSITY HOSPITAL | Beckett Street, Leeds, West Yorkshire | LS9 7TF |
| Mr J A A Archbold | Archbold | DOWNE HOSPITAL | 9A Pound Lane, Down Patrick, Co Downe | BT30 6JA |
| Dr D Ash | Ash | COOKRIDGE HOSPITAL | Hospital Lane, Leeds | LS16 6QB |
| Dr Richard F U Ashford | Ashford | MOUNT VERNON CENTRE FOR CANCER TREATMENT | Rickmansworth Road, Northwood, Middlesex | HA6 2RN |
| Mohammed Ashraf | Ashraf | KING'S MILL HOSPITAL | Mansfield Road , Sutton-In-Ashfield, Nottinghamshire, | NG17 4JL |
| Dr Aslam | Aslam | BEDFORD HOSPITAL NORTH WING | Kimbolton Road, Bedford | MK40 2AW |
| Dr Roger Avill | Avill | DONCASTER ROYAL INFIRMARY (BASSETLAW HOSPITAL) | Armthorpe Road, Doncaster | DN2 5LT |
| Ninaad Awsare | Awsare | COUNTESS OF CHESTER HOSPITAL | The Countess Of Chester Health Park , Chester, Cheshire | CH2 1HJ |
| Faisal Azam | Azam | WREXHAM MAELOR HOSPITAL | Croesnewydd Road, Wrexham | LL13 7TD |
| Dr A Azzabi | Azzabi | FREEMAN HOSPITAL | Northern Centre for Cancer Care, Freeman Road, High Heaton, Newcastle upon Tyne | NE7 7DN |
| Mr David Badenoch | Badenoch | HARLEY STREET CONSULTING ROOMS | 101 Harley Street, London | W1G 6AH |
| Dr Amit Bahl | Bahl | BRISTOL ROYAL INFIRMARY | Marlborough Street, Bristol | BS2 8HW |
| Mr M J Bailey | Bailey | ST GEORGE'S HOSPITAL | Blackshaw Road, Tooting, London | SW17 0QT |
| Mrs Karen Bailey | Bailey | UNIVERSITY HOSPITAL OF WALES | Heath Park, Cardiff, Wales | CF14 4XW |
| Mr Andrew J Ball | Ball | SOUTHEND UNIVERSITY HOSPITAL | Prittlewell Chase, Westcliff on Sea, Essex | SS0 0RY |
| Dr David Ballesteros | Ballesteros | LINCOLN COUNTY HOSPITAL | Greetwell Road , Lincoln, Lincolnshire | LN2 5QY |
| Mr G Banerjee | Banerjee | IPSWICH HOSPITAL | Heath Road, Ipswich, Suffolk | IP4 5PD |
| Dr N Barber | Barber | KING'S COLLEGE HOSPITAL | Denmark Hill, London | SE5 9RS |
| Dr Jim Barber | Barber | VELINDRE HOSPITAL | Whitchurch, Cardiff, Wales | CF14 2TL |
| Dr Baria | Baria | PILGRIM HOSPITAL | Sibsey Road, Boston, Lincs | PE21 9QS |
| Mr Douglas G Barnes | Barnes | NORTH MANCHESTER GENERAL HOSPITAL | Delauneys Road, Crumpsall, Manchester | M8 5RB |
| Mr J Bashir | Bashir | CUMBERLAND INFIRMARY | Cumberland Infirmary, Newtown Road, Carlisle, Cumbria | CA2 7HY |
| Mr Pradip Basu | Basu | LINCOLN COUNTY HOSPITAL | Greetwell Road, Lincoln | LN2 5QY |
| Mr Christopher A Bates | Bates | ROYAL GWENT HOSPITAL | Cardiff Road, Newport,Gwent | NP20 2UB |
| Dr N A Bax | Bax | EASTBOURNE DISTRICT GENERAL HOSPITAL | King's Drive, Eastbourne, East Sussex | BN21 2UD |

| | | | | |
|---|---|---|---|---|
| Mr D Baxter-Smith | Baxter-Smith | KIDDERMINSTER HOSPITAL | Bewdley Road , Kidderminster, Worcestershire | DY11 6RJ |
| Mr Amar Bdesha | Bdesha | WYCOMBE GENERAL HOSPITAL | Queen Alexander Rd, High Wycombe, Buckinghamshire | HP11 2TT |
| Mr Christopher J M Beacock | Beacock | ROYAL SHREWSBURY HOSPITAL | Mytton Oak Road, Shrewsbury | SY3 8XQ |
| Professor Ronald P Beaney | Beaney | ST THOMAS'S HOSPITAL | Lambeth Palace Road, London | SE1 7EH |
| Mr Ralph Beard | Beard | WORTHING HOSPITAL | Lyndhurst Road, Worthing, West Sussex | BN11 2DH |
| Mr John D Beatty | Beatty | NORTHAMPTON GENERAL HOSPITAL | Billing Rd, Northampton | NN1 5BD |
| Mr Rupert Beck | Beck | GREAT WESTERN HOSPITAL | Marlborough Road, Swindon, Wilts | SN3 6BB |
| Ms Gail Beese | Beese | ROYAL GWENT HOSPITAL | Cardiff Road, Newport,Gwent | NP9 2UB |
| Dr Sharon Beesley | Beesley | MAIDSTONE HOSPITAL | Hermitage Lane, Kent | ME16 9QQ |
| Mr C Richard W Bell | Bell | NORTHAMPTON GENERAL HOSPITAL | Billing Rd, Northampton | NN1 5BD |
| Mr James Bellringer | Bellringer | CHARING CROSS HOSPITAL | Fulham Palace Road, London | W6 8RF |
| Dr Richard Benson | Benson | PETERBOROUGH DISTRICT HOSPITAL | Thorper Road, Peterborough, Cambridgeshire | PE3 6DA |
| Dr Beresford | Beresford | ROYAL UNITED HOSPITAL BATH | Combe Park, Bath | BA1 3AG |
| Mr Christopher R A Bevis | Bevis | BLACKPOOL VICTORIA HOSPITAL | Whinney Heys Road, Blackpool, Lancashire | FY3 8NR |
| Neeraj Bhalla | Bhalla | THE CLATTERBRIDGE CANCER CENTRE | Clatterbridge Rd, Bebington, Wirral | CH63 4JY |
| Dr Rajanee Bhana | Bhana | QUEEN ELIZABETH HOSPITAL | Mindelsohn Way, Edgbaston, Birmingham | B15 2TH |
| Mr S Bhanot | Bhanot | KING GEORGE HOSPITAL | Barley Lane, Goodmayes, Ilford | IG3 8YB |
| Mr Tahir Rasool Bhat | Bhat | MEDWAY MARITIME HOSPITAL | Windmill Road, Gillingham | ME7 5NY |
| Dr A Bhatnagar | Bhatnagar | SALISBURY DISTRICT HOSPITAL | Odstock Road, Salisbury, Wilts | SP2 8BJ |
| Mr R I Bhatt | Bhatt | QUEEN ELIZABETH HOSPITAL | Mindelsohn Way, Edgbaston, Birmingham | B15 2TH |
| Mr Brian Birch | Birch | ROYAL SOUTH HANTS HOSPITAL | The Wessex Rt Centre,St Mary's Road, Southampton | SO14 0YG |
| Dr Alison Birtle | Birtle | ROYAL PRESTON HOSPITAL | Sharoe Green Lane North, Fulwood, Preston, Lancashire | PR2 9HT |
| Mr M Bishop | Bishop | NOTTINGHAM CITY HOSPITAL | Hucknall Road, Nottingham | NG5 1PB |
| Mr C Shekhar Biyani | Biyani | PINDERFIELDS HOSPITAL | Aberford Road, Wakefield, West Yorkshire | WF1 4DG |
| Anthony Blacker | Blacker | UNIVERSITY HOSPITAL (COVENTRY) | Clifford Bridge Road, Walsgrave, Coventry | CV2 2DX |
| Mr A R E Blacklock | Blacklock | UNIVERSITY HOSPITAL OF COVENTRY AND WARWICKSHIRE | Clifford Bridge Road, Walsgrave, Coventry | CV2 2DX |
| Miss Rosemary Blades | Blades | ROYAL PRESTON HOSPITAL | Sharoe Green Lane North, Fulwood, Preston, Lancashire | PR2 9HT |
| Mr Christopher Blake | Blake | ROYAL CORNWALL HOSPITAL (TRELISKE) | Treliske, Truro, Cornwall | TR1 3LJ |
| Mr Benedict Blake-James | Blake-James | YORK HOSPITAL | Wiggington Road, York, Yorkshire | YO31 8HE |
| Dr Peter Bliss | Bliss | TORBAY HOSPITAL | Lawes Bridge, Torquay, Devon | TQ2 7AA |
| Dr David J Bloomfield | Bloomfield | ROYAL SUSSEX COUNTY HOSPITAL | Eastern Road, Brighton, East Sussex | BN2 5BE |
| Miss S Boddy | Boddy | NEW CROSS HOSPITAL | Wednesfield Road, Wolverhampton, West Midlands | WV10 0QP |
| Ashok Bojwani | Bojwani | KING'S MILL HOSPITAL | Mansfield Road , Sutton-In-Ashfield, Nottinghamshire, | NG17 4JL |
| Professor C M Booth | Booth | COLCHESTER GENERAL HOSPITAL | Turner Road, Colchester, Essex | CO4 5JL |
| Mr Matthieu Bordenave | Bordenave | CUMBERLAND INFIRMARY | Cumberland Infirmary, Newtown Road, Carlisle, Cumbria | CA2 7HY |
| Mr Pradeep Bose | Bose | MORRISTON HOSPITAL | Morriston, Swansea, Wales | SA6 6NL |
| Dr Michael C Bott | Bott | FRIMLEY PARK HOSPITAL | Portsmouth Road, Frimley, Camberley | GU16 7UJ |
| Dr David Bottomley | Bottomley | COOKRIDGE HOSPITAL | Hospital Lane, Leeds | LS16 6QB |
| Mr Nigel R Boucher | Boucher | CHESTERFIELD ROYAL HOSPITAL | Calow, Chesterfield, Derbyshire | S44 5BL |
| Dr J Bowen | Bowen | WORCESTERSHIRE ROYAL HOSPITAL | Charles Hastings Way, Worcs | WR5 1DD |
| Dr Mark Bower | Bower | CHELSEA & WESTMINSTER HOSPITAL | 368 Fulham Road, London | SW10 9RH |
| Mr W G Bowsher | Bowsher | ROYAL GWENT HOSPITAL | Cardiff Road, Newport,Gwent | NP9 2UB |
| Mr P J R Boyd | Boyd | ST HELIER HOSPITAL | Wrythe Green Lane,Carshalton, Surrey | SM5 1AA |
| Mr F James Bramble | Bramble | ROYAL BOURNEMOUTH HOSPITAL | Castle Lane East, Bournemouth, Dorset | BH7 7DW |
| Mr Simon F Brewster | Brewster | CHURCHILL HOSPITAL | Old Road, Headington, Oxford | OX3 7LJ |
| Dr Robert Brierly | Brierly | THE IPSWICH HOSPITAL NHS TRUST | Heath Road, Ipswich, Suffolk | IP4 5PD |
| Mr Tim Briggs | Briggs | BARNET GENERAL HOSPITAL | Wellhouse Lane, Barnet, Herts | EN5 3DJ |
| Mr John Britton | Britton | ST RICHARD'S HOSPITAL | Spitalfields Lane, Chichester, West Sussex | PO19 6SE |
| Dr Cathryn Brock | Brock | CHELSEA AND WESTMINSTER HOSPITAL | 370 Fulham Road, London | SW10 9RH |
| Dr Sue Brock | Brock | ROYAL BOURNEMOUTH HOSPITAL | Castle Lane East, Bournemouth, Dorset | BH7 7DW |
| Mr Stephen Bromage | Bromage | TAMESIDE GENERAL HOSPITAL | Fountain Street, Ashton-under-Lyne | OL6 9RW |
| Mr Richard Brough | Brough | TAMESIDE GENERAL HOSPITAL | Fountain Street, Ashton-Under-Lyne | OL6 9RW |
| Dr Richard Brown | Brown | ROYAL BERKSHIRE HOSPITAL | London Road, Reading, Berkshire | RG1 5AN |
| Mr Stephen Brown | Brown | STEPPING HILL HOSPITAL | Poplar Grove, Hazel Grove, Stockport | SK2 7JE |
| Mr Richard Brown | Brown | WEXHAM PARK HOSPITAL | Wexham street, Slough, Berkshire | SL2 4HL |
| Mr Tony J Browning | Browning | PINDERFIELDS HOSPITAL | Aberford Road, Wakefield, West Yorkshire | WF1 4DG |
| Mr N Bryan | Bryan | HUDDERSFIELD ROYAL INFIRMARY | Lindley, Huddersfield | HD3 3EA |
| Mr Neil A Burgess | Burgess | NORFOLK & NORWICH UNIVERSITY HOSPITAL | Colney Lane, Norwich | NR4 7UZ |
| Mr Nicholas Burns-Cox | Burns-Cox | MUSGROVE PARK HOSPITAL | Mugrove Park, Taunton | TA1 5DA |
| Mr Paul C Butterworth | Butterworth | LEICESTER GENERAL HOSPITAL | Gwendolen Road, Leicester | LE5 4PW |
| Dr Mick Button | Button | VELINDRE HOSPITAL | Whitchurch, Cardiff, Wales | CF14 2TL |

| | | | | |
|---|---|---|---|---|
| Mr D Cahill | Cahill | GUY'S HOSPITAL | St Thomas Street, London | SE1 9RT |
| Mr P S Callaghan | Callaghan | CONQUEST HOSPITAL | The Ridge, St Leonards on Sea, Hastings, East Sussex | TN37 7RD |
| Mr John Calleary | Calleary | NORTH MANCHESTER GENERAL HOSPITAL | Delauneys Road, Crumpsall, Manchester | M8 5RB |
| Dr M Calleja | Calleja | QUEEN ELIZABETH HOSPITAL KING'S LYNN | Gayton Road, King's Lynn, Norfolk | PE30 4ET |
| Dr Frances Calman | Calman | ST THOMAS'S HOSPITAL | Lambeth Palace Road, London | SE1 7EH |
| Dr Philip Camilleri | Camilleri | NORTHAMPTON GENERAL HOSPITAL | Billing Rd, Northampton | NN1 5BD |
| Mr Alister Campbell | Campbell | SALISBURY DISTRICT HOSPITAL | Odstock Road, Salisbury, Wilts | SP2 8BJ |
| Miss Andrea Cannon | Cannon | MUSGROVE PARK HOSPITAL | Mugrove Park, Taunton | TA1 5DA |
| Dr Lisa Margaret Capaldi | Capaldi | KIDDERMINSTER HOSPITAL | Bewdley Road , Kidderminster, Worcestershire | DY11 6RJ |
| Dr Dawn M Carnell | Carnell | UNIVERSITY COLLEGE HOSPITAL | 235 Euston Rd, Fitzrovia, London | NW1 2BU |
| Mr T W Carr | Carr | SOUTHEND UNIVERSITY HOSPITAL | Prittlewell Chase, Westcliff on Sea, Essex | SS0 0RY |
| Dr Carser | Carser | CRAIGAVON AREA HOSPITAL | 68 Lurgan Road, Portadown, Craigavon | BT63 5QQ |
| Mr Simon Carter | Carter | CHARING CROSS HOSPITAL | Fulham Palace Road, London | W6 8RF |
| Mr Charles J M Carter | Carter | ROYAL BOURNEMOUTH HOSPITAL | Castle Lane East, Bournemouth, Dorset | BH7 7DW |
| Dr Adam C Carter | Carter | ROYAL GWENT HOSPITAL | Cardiff Road, Newport,Gwent | NP9 2UB |
| Dr Hannah Casey | Casey | BROOMFIELD HOSPITAL | Court Road, Broomfield, Chelmsford, Essex | CM1 5ET |
| Dr Bruce M Castle | Castle | PRINCESS ANNE HOSPITAL | Coxford Rd, Southampton, Hants | SO16 5YA |
| Mr David Chadwick | Chadwick | JAMES COOK UNIVERSITY HOSPITAL | Department of Urology, Marton Road, Middlesbrough | TS4 3BW |
| Dr Eliot Chadwick | Chadwick | NOTTINGHAM CITY HOSPITAL | Hucknall Road, Nottingham | NG5 1PB |
| Mr Rohit Chahal | Chahal | PINDERFIELDS HOSPITAL | Aberford Road, Wakefield, West Yorkshire | WF1 4DG |
| Dr P Chakraborti | Chakraborti | QUEENS HOSPITAL BURTON | Belvedere Road, Burton-On-Trent, Derbyshire | DE13 0RB |
| Dr Prabir Chakraborti | Chakraborti | ROYAL DERBY HOSPITAL | Derbyshire Royal Infirmary, London Road, Derby | DE1 2QY |
| Dr Andrew Chan | Chan | GEORGE ELIOT HOSPITAL | College Street, Nuneaton, Warks | CV10 7BL |
| Mr Chaplin | Chaplin | UNIVERSITY HOSPITAL OF NORTH TEES | Hardwick, Stockton on Tees | TS19 8PE |
| Mr Chappell | Chappell | WORTHING HOSPITAL | Worthing Hospital, Worthing, Sussex | BN11 2DH |
| Mr C Charig | Charig | EPSOM GENERAL HOSPITAL | Dorking Road, Epsom, Surrey | KT18 7EG |
| Natalie Charnley | Charnley | ROYAL BLACKBURN HOSPITAL | Haslingdon Road, Blackburn | BB2 3HH |
| Mr Terng Fong Chen | Chen | WORCESTERSHIRE ROYAL HOSPITAL | Charles Hastings Way, Worcester | WR5 1DD |
| Dr Anula D Chetiyawardana | Chetiyawardana | MANOR HOSPITAL | Moat Road, Walsall, West Midlands | WS2 9PS |
| Mr Christopher Chilton | Chilton | DERBY CITY GENERAL HOSPITAL | Uttoxeter Road, Derby | DE22 3NE |
| Mr F I Chinegwundoh | Chinegwundoh | ST BARTHOLOMEW'S HOSPITAL | West Smithfield, London | EC1A 7BE |
| Dr Irene Chong | Chong | BARNET GENERAL HOSPITAL | Wellhouse Lane, Barnet, Herts | EN5 3DJ |
| Dr Ananya Choudhury | Choudhury | ROYAL OLDHAM HOSPITAL | 1st Floor, J Block, Rochdale Road, Oldham | OL1 2JH |
| Mr Wai-Man Chow | Chow | NORTH MANCHESTER GENERAL HOSPITAL | Delaunays Road, Crumpsall, Manchester | M8 5RB |
| Mr Timothy J Christmas | Christmas | CHARING CROSS HOSPITAL | Fulham Palace Road, London | W6 8RF |
| Dr Mark J Churn | Churn | NEW CROSS HOSPITAL | Wednesfield Road, Wolverhampton, West Midlands | WV10 0QP |
| Mr Noel W Clarke | Clarke | HOPE HOSPITAL | Stott Lane, Salford | M6 8HD |
| Mr Jorge Clavijo-Eisele | Clavijo-Eisele | DIANA PRINCESS OF WALES HOSPITAL | Scartho Road, Grimsby | DN33 2BA |
| Mr Andrew Michael Cliff | Cliff | ARROWE PARK HOSPITAL | Arrowe Park Road, Upton , Wirral, Merseyside | CH49 5PE |
| Ms O Clyne | Clyne | NEW CROSS HOSPITAL | Wednesfield Road, Wolverhampton, West Midlands | WV10 0QP |
| Dr M Coe | Coe | CLATTERBRIDGE CENTRE FOR ONCOLOGY | Clatterbridge Rd, Bebington, Wirral | CH63 4JY |
| Mr N P Cohen | Cohen | ABERDEEN ROYAL INFIRMARY | Foresterhill, Aberdeen | AB25 22N |
| Mr C Coker | Coker | ROYAL SUSSEX COUNTY HOSPITAL | Eastern Road, Brighton, East Sussex | BN2 5BE |
| Dr Trevor Cole | Cole | BIRMINGHAM WOMEN'S HOSPITAL | Clinical Genetics Unit, Edgbaston, Birmingham | B15 2TG |
| Dr David J Cole | Cole | CHURCHILL HOSPITAL | Old Road, Headington, Oxford | OX3 7LJ |
| Mr O Cole | Cole | NOTTINGHAM CITY HOSPITAL | Hucknall Road, Nottingham | NG5 1PB |
| Mr Gerald Collins | Collins | STEPPING HILL HOSPITAL | Poplar Grove, Hazel Grove, Stockport | SK2 7JE |
| Dr Matthew Collinson | Collinson | ROYAL CORNWALL HOSPITAL | Treliske, Truro, Cornwall | TR1 3LJ |
| Mr I Conn | Conn | ABERDEEN ROYAL INFIRMARY | Foresterhill, Aberdeen | AB25 22N |
| Dr C Connell | Connell | CHURCHILL HOSPITAL | Old Road, Headington, Oxford | OX3 7LJ |
| Dr Conroy | Conroy | ROYAL OLDHAM HOSPITAL | Rochdale Road, Oldham, Greater Manchester | OL1 2JH |
| Dr Audrey Cook | Cook | CHELTENHAM GENERAL HOSPITAL | Sandford Road, Cheltenham, Gloucester | GL53 7AN |
| Mr Peter Cooke | Cooke | NEW CROSS HOSPITAL | Wednesfield Road, Wolverhampton, West Midlands | WV10 0QP |
| Mr Graeme Cooksey | Cooksey | CASTLE HILL HOSPITAL | Castle Road, Cottingham, East Yorkshire | HU16 5JQ |
| Mr L Coombs | Coombs | SCUNTHORPE GENERAL HOSPITAL | Cliff Gardens, Scunthorpe, N Lincolnshire | DN15 7BH |
| Mr Robert F Copland | Copland | KINGS OAK PRIVATE HOSPITAL | Chase Farm (North Side), The Ridgeway, Enfield | EN2 8SD |
| Mr Andrew J Cornaby | Cornaby | DORSET COUNTY HOSPITAL | Williams Avenue, Dorchester | DT1 2JY |
| Mr P A Cornford | Cornford | ROYAL LIVERPOOL UNIVERSITY HOSPITAL | Prescot Street, Liverpool | L7 8XP |
| Mr Corolis | Corolis | SOUTHMEAD HOSPITAL | Westbury-on-Trym, Bristol, Somerset | BS10 5NB |
| Mr John Corr | Corr | COLCHESTER GENERAL HOSPITAL | Turner Road, Colchester, Essex | CO4 5JL |
| Mr C B Costello | Costello | NORTH MANCHESTER GENERAL HOSPITAL | Delauneys Road, Crumpsall, Manchester | M8 5RB |

| | | | | |
|---|---|---|---|---|
| Mrs N Coull | Coull | KINGSTON HOSPITAL | Galsworthy Road, Kingston-upon-Thames, Surrey | KT2 7QB |
| Dr Richard Cowan | Cowan | CHRISTIE HOSPITAL | Wilmslow Road, Withington, Manchester | M20 4BX |
| Mr Robert Cox | Cox | ROYAL CORNWALL HOSPITAL | Treliske, Truro, Cornwall | TR1 3LJ |
| Dr C Coyle | Coyle | COOKRIDGE HOSPITAL | Hospital Lane, Leeds | LS16 6QB |
| Simon Crabb | Crabb | SOUTHAMPTON UNIVERSITY HOSPITAL | Tremona Road, Southampton | SO16 6YD |
| Mr Jeremy Crew | Crew | CHURCHILL HOSPITAL | Old Road, Headington, Oxford | OX3 7LJ |
| Mr John C Crisp | Crisp | WATFORD GENERAL HOSPITAL | Vicarage Road, Watford, Hertfordshire | WD1 8HB |
| Dr W Cross | Cross | ST JAMES' UNIVERSITY HOSPITAL | Beckett Street, Leeds, West Yorkshire | LS9 7TF |
| Mr W Cross | Cross | ST JAMES' UNIVERSITY HOSPITAL | Beckett Street, Leeds, West Yorkshire | LS9 7TF |
| Dr Dorthe Cruger | Cruger | BIRMINGHAM WOMEN'S HOSPITAL | Clinical Genetics Unit, Edgbaston, Birmingham | B15 2TG |
| Mr Michael Crundwell | Crundwell | ROYAL DEVON & EXETER HOSPITAL (WONFORD) | Barrack Rd, Exeter, Devon | EX2 5DW |
| Mr Malcolm Crundwell | Crundwell | ROYAL DEVON AND EXETER HOSPITAL | Barrack Rd, Exeter, Devon | EX2 5DW |
| John Cumming | Cumming | CUMBERLAND INFIRMARY | Cumberland Infirmary, Newtown Road, Carlisle, Cumbria | CA2 7HY |
| Mr Cummings | Cummings | PRINCESS ANNE HOSPITAL | Coxford Rd, Southampton, Hants | SO16 5YA |
| Mr Nazeer Dahar | Dahar | GRANTHAM & DISTRICT HOSPITAL | 101 Manthorpe Road, Grantham, Lincs | NG31 8DG |
| Dr Nicola Dallas | Dallas | WEXHAM PARK HOSPITAL | Wexham street, Slough, Berkshire | SL2 4HL |
| Dr Francis N Daniel | Daniel | DERRIFORD HOSPITAL | Derriford Road, Plymouth | PL6 8DH |
| Mr J Darrad | Darrad | DONCASTER ROYAL INFIRMARY | Doncaster Royal Infirmary, C Block, Doncaster Royal Infirmary, Armthorpe Road, Doncaster | DN2 5LT |
| Mr Pallon Daruwala | Daruwala | GRANTHAM & DISTRICT HOSPITAL | 101 Manthorpe Road, Grantham, Lincs | NG31 8DG |
| Mr Gautam Das | Das | CROYDON UNIVERSITY HOSPITAL | Research Office, 1st Floor, Woodcroft Wing, Croydon University Hospital, Croydon | CR7 7YE |
| Mr Shibendra Datta | Datta | UNIVERSITY HOSPITAL OF WALES | Heath Park, Cardiff, Wales | CF14 4XW |
| Mr Dauleh | Dauleh | ROYAL ALBERT EDWARD INFIRMARY | Wigan Lane, Wigan, Lancashire | WN1 2NN |
| Dr S Davidson | Davidson | MACCLESFIELD DISTRICT GENERAL HOSPITAL | Victoria Road, Macclesfield, Cheshire | SK10 3BL |
| Dr Joseph Davies | Davies | ROYAL BOURNEMOUTH HOSPITAL | Castle Lane East, Bournemouth, Dorset | BH7 7DW |
| Mr Owen W Davison | Davison | KETTERING GENERAL HOSPITAL | Rothwell Road, Kettering, Northants | NN16 8UZ |
| Dr Davola | Davola | PRINCESS ALEXANDRA HOSPITAL | Cancer Services, Galen House, Hamstel Road, Harlow, Essex | CM20 1QX |
| Mr Guy Dawkins | Dawkins | PRINCESS ROYAL UNIVERSITY HOSPITAL | Farnborough Common, Orpington, Kent | BR6 8ND |
| Mr Chris Dawson | Dawson | EDITH CAVELL HOSPITAL | Bretton Gate, Peterborough, Cambridgeshire | PE3 9GZ |
| Mr Alan R De Bolla | De Bolla | WREXHAM MAELOR HOSPITAL | Croesnewydd Road, Wrexham | LL13 7TD |
| Professor David Dearnaley | Dearnaley | ROYAL MARSDEN HOSPITAL | Fulham Road, London | SW3 6JJ |
| Mr Ken M Desai | Desai | UNIVERSITY HOSPITAL OF COVENTRY AND WARWICKSHIRE | Clifford Bridge Road, Walsgrave, Coventry | CV2 2DX |
| Dr George P Deutsch | Deutsch | ROYAL SUSSEX COUNTY HOSPITAL | Eastern Road, Brighton, East Sussex | BN2 5BE |
| Mr John Dick | Dick | KINGSTON HOSPITAL | Galsworthy Road, Kingston-upon-Thames, Surrey | KT2 7QB |
| Mr Andrew J Dickinson | Dickinson | DERRIFORD HOSPITAL | Derriford Road, Plymouth | PL6 8DH |
| Dr Jeanette Dickson | Dickson | MOUNT VERNON CENTRE FOR CANCER TREATMENT | Rickmansworth Road, Northwood, Middlesex | HA6 2RN |
| Mr Panagiotis Dimopoulos | Dimopoulos | SOUTHEND HOSPITAL | Westcliffe-on-Sea, Essex | SS0 0RY |
| Dr Din | Din | CHESTERFIELD ROYAL HOSPITAL | Calow, Chesterfield, Derbyshire | S44 5BL |
| Mr Michael Dinneen | Dinneen | CHELSEA & WESTMINSTER HOSPITAL | 369 Fulham Road, London | SW10 9RH |
| Dr Sanjay Dixit | Dixit | SCUNTHORPE GENERAL HOSPITAL | Cliff Gardens, Scunthorpe, N Lincolnshire | DN15 7BH |
| Dr H Jane Dobbs | Dobbs | GUY'S HOSPITAL | St Thomas Street, London | SE1 9RT |
| Mr A Doble | Doble | ADDENBROOKE'S HOSPITAL | Hills Road, Cambridge | CB2 2QQ |
| Dr David Dodds | Dodds | BEATSON ONCOLOGY CENTRE | Western Infirmary, Dumbarton Road, Glasgow | G11 6NT |
| Mr Alan Doherty | Doherty | QUEEN ELIZABETH HOSPITAL | Mindelsohn Way, Edgbaston, Birmingham | B15 2TH |
| Mr P Donaldson | Donaldson | IPSWICH HOSPITAL | Heath Road, Ipswich, Suffolk | IP4 5PD |
| Mr John Donohue | Donohue | THE MAIDSTONE HOSPITAL | Hermitage Lane, Maidstone, Kent | ME16 9QQ |
| Dr M Dooldeniya | Dooldeniya | SOUTHEND GENERAL HOSPITAL | Westcliffe-on-Sea, Essex | SS0 0RY |
| Dr S Fiona Douglas | Douglas | INSTITUTE OF HUMAN GENETICS INTERNATIONAL CENTRE FOR LIFE | Central Parkway, Newcastle upon Tyne | NE1 3BZ |
| Mr Drake | Drake | SOUTHMEAD HOSPITAL | Westbury-on-Trym, Bristol, Somerset | BS10 5NB |
| Dr Gill M Duchesne | Duchesne | UNIVERSITY COLLEGE HOSPITAL | 242 Euston Rd, Fitzrovia, London | NW1 2BU |
| Mr Peter Duffy | Duffy | ROYAL LANCASTER INFIRMARY | Ashton Road, Lancaster, Lancashire | LA1 4RP |
| Mr Michael Dunn | Dunn | NOTTINGHAM NUFFIELD HOSPITAL | 748 Mansfield Road, Woodthorpe, Nottingham | NG5 3FZ |
| Mr W D Dunsmuir | Dunsmuir | ST PETER'S HOSPITAL | Guildford Road, Lyne, Chertsey | KT16 0PZ |
| Dr Sajid K Durrani | Durrani | BRONGLAIS GENERAL HOSPITAL | Caradog Road, Aberystwyth | SY23 1ER |
| Mr Johnathan Dyer | Dyer | SOUTHAMPTON GENERAL HOSPITAL | Tremona Road , Southampton, Hampshire | SO16 6YD |
| Mr Alan C Eaton | Eaton | QUEEN ELIZABETH HOSPITAL KINGS LYNN | Gayton Road, King's Lynn, Norfolk | PE30 4ET |
| Professor Diane Eccles | Eccles | PRINCESS ANNE HOSPITAL | Coxford Rd, Southampton, Hants | SO16 5YA |
| Mr B Eddy | Eddy | KENT AND CANTERBURY HOSPITAL | Ethelbert Road, Canterbury, Kent | CT1 3NG |
| Mr C D Eden | Eden | FRIMLEY PARK HOSPITAL | Portsmouth Road, Frimley, Camberley | GU16 7UJ |
| Mr J Edwards | Edwards | BRONGLAIS GENERAL HOSPITAL | Caradog Road, Aberystwyth | SY23 1ER |
| Mr Jeremy Elkabir | Elkabir | NORTHWICK PARK HOSPITAL | Watford Road, Harrow | HA1 3UJ |
| Dr P Tony Elliott | Elliott | CHRISTIE HOSPITAL | Wilmslow Road, Withington, Manchester | M20 4BX |

| | | | | |
|---|---|---|---|---|
| Mr B W Ellis | Ellis | ASHFORD HOSPITAL | London Road, Stanwell, Ashford | TW15 3AA |
| Dr R Ellis | Ellis | ROYAL CORNWALL HOSPITAL | Treliske, Truro, Cornwall | TR1 3LJ |
| Dr A El-Modir | El-Modir | QUEEN ELIZABETH HOSPITAL | Mindelsohn Way, Edgbaston, Birmingham | B15 2TH |
| Mr Andrew W S Elves | Elves | ROYAL SHREWSBURY HOSPITAL | Mytton Oak Road, Shrewsbury | SY3 8XQ |
| Dr Christine Elwell | Elwell | NORTHAMPTON GENERAL HOSPITAL | Billing Rd, Northampton | NN1 5BD |
| Mr Mark Emberton | Emberton | UNIVERSITY COLLEGE HOSPITAL | 236 Euston Rd, Fitzrovia, London | NW1 2BU |
| Dr Louise Emmerson | Emmerson | GLAN CLWYD HOSPITAL | Bodelwyddan, Rhyl, Wales | LL18 5UJ |
| Mr Roland C D England | England | KETTERING GENERAL HOSPITAL | Rothwell Road, Kettering, Northants | NN16 8UZ |
| Oparaku Umez-Eronini | Eronini | CUMBERLAND INFIRMARY | Cumberland Infirmary, Newtown Road, Carlisle, Cumbria | CA2 7HY |
| Mr R D Errington | Errington | CLATTERBRIDGE CENTRE FOR ONCOLOGY | Clatterbridge Rd, Bebington, Wirral | CH63 4JY |
| Professor D Gareth Evans | Evans | CHRISTIE HOSPITAL | Wilmslow Road, Withington, Manchester | M20 4BX |
| Dr Everest | Everest | ROYAL UNITED HOSPITAL BATH | Combe Park, Bath | BA1 3AG |
| Dr Alison Falconer | Falconer | CHARING CROSS HOSPITAL | Fulham Palace Road, London | W6 8RF |
| Mr Derek Fawcett | Fawcett | ROYAL BERKSHIRE HOSPITAL | London Road, Reading, Berkshire | RG1 5AN |
| Dr C Featherston | Featherston | BEATSON ONCOLOGY CENTRE | Western Infirmary, Dumbarton Road, Glasgow | G11 6NT |
| Dr Carolyn J Featherstone | Featherstone | BEATSON ONCOLOGY CENTRE | Western Infirmary, Dumbarton Road, Glasgow | G11 6NT |
| Mr P Featherstone | Featherstone | UNIVERSITY HOSPITAL OF WALES | Heath Park, Cardiff, Wales | CF14 4XW |
| Mr Jeremy Feggetter | Feggetter | BERWICK INFIRMARY | Berwick Upon Tweed, Northumbria | TD15 1LT |
| Dr Fenn | Fenn | WITHYBUSH GENERAL HOSPITAL | Ward 10, Fishguard Road, Haverford West, Pembrokeshire | SA61 2PZ |
| Mr S Fenugopal | Fenugopal | CHESTERFIELD ROYAL HOSPITAL | Calow, Chesterfield | S44 5BL |
| Dr C Ferguson | Ferguson | WESTON PARK HOSPITAL | Whitham Road, Sheffield, South Yorkshire | S10 2SJ |
| Dr D Fermont | Fermont | NORTHWICK PARK HOSPITAL | Watford Road, Harrow | HA1 3UJ |
| Mr Michael Ferro | Ferro | HUDDERSFIELD ROYAL INFIRMARY | Lindley, Huddersfield | HD3 3EA |
| Mr Flavin | Flavin | WREXHAM MAELOR HOSPITAL | Croesnewydd Road, Wrexham | LL13 7TD |
| Mr Matthew Fletcher | Fletcher | ROYAL SUSSEX COUNTY HOSPITAL | Eastern Road, Brighton, East Sussex | BN2 5BE |
| Dr A Folkes | Folkes | EAST SURREY HOSPITAL | Canada Ave, Redhill, Surrey | RH1 5RH |
| Mr Trevor F Ford | Ford | MAIDSTONE HOSPITAL | Hermitage Lane, Barming, Kent | ME16 9QQ |
| Mr Paul W Foster | Foster | YEOVIL DISTRICT HOSPITAL | Higher Kingston, Yeovil, Somerset | BA21 4AT |
| Dr Kevin N Franks | Franks | ST JAMES' UNIVERSITY HOSPITAL | Beckett Street, Leeds, West Yorkshire | LS9 7TF |
| Miss Jane French | French | THE ROYAL GLAMORGAN HOSPITAL | Ynysmaerdy, Llantrisant | CF72 8XR |
| Dr Olivera Frim | Frim | ROYAL UNITED HOSPITAL BATH | Combe Park, Bath | BA1 3AG |
| Dr Joanna Gale | Gale | QUEEN ALEXANDRA HOSPITAL, PORTSMOUTH | Cosham, Portsmouth | P06 3LY |
| Mr Christopher Gallegos | Gallegos | ROYAL UNITED HOSPITAL BATH | Combe Park, Bath | BA1 3NG |
| Mr. Hosea B. Gana | Gana | ST HELENS HOSPITAL | Marshalls Cross Road, St Helens | WA9 3DA |
| Mr S. Ganta | Ganta | MANOR HOSPITAL | Moat Road, Walsall, West Midlands | WS2 9PS |
| Mr James S Gelister | Gelister | BARNET GENERAL HOSPITAL | Wellhouse Lane, Barnet, Herts | EN5 3DJ |
| Dr Ghana | Ghana | WITHYBUSH GENERAL HOSPITAL | Ward 10, Fishguard Road, Haverford West, Pembrokeshire | SA61 2PZ |
| Mr Maneesh Ghei | Ghei | THE WHITTINGTON HOSPITAL | Magdala Avenue, London | N19 5NF |
| Dr Stephanie Gibbs | Gibbs | QUEEN'S HOSPITAL | Rom Valley Way, Romford, Essex | RM7 0AG |
| Mr Hugh Gilbert | Gilbert | CHELTENHAM GENERAL HOSPITAL | Sandford Road, Cheltenham, Gloucester | GL53 7AN |
| Mr Johnathan Gill | Gill | HARROGATE DISTRICT HOSPITAL | Lancaster Park Road, Harrogate | HG2 7SX |
| Mr David Gillatt | Gillatt | SOUTHMEAD HOSPITAL | Westbury-on-Trym, Bristol, Somerset | BS10 5NB |
| Dr John Glaholm | Glaholm | GOOD HOPE HOSPITAL | Rectory Road, Sutton Coldfield, West Midlands | B75 7RR |
| Mr Jonathan M Glass | Glass | ST THOMAS'S HOSPITAL | Lambeth Palace Road, London | SE1 7EH |
| Dr Hilary Glen | Glen | UNIVERSITY HOSPITAL AYR | Dalmellington Road, Ayr, Scotland | KA6 6DX |
| Mr James Glenister | Glenister | FINCHLEY MEMORIAL HOSPITAL | Granville Road, North Finchley, London | N12 0JE |
| Dr Chee Goh | Goh | EAST SURREY HOSPITAL | Canada Ave, Redhill, Surrey | RH1 5RH |
| Dr Naomi Goldstraw | Goldstraw | ROYAL DEVON & EXETER HOSPITAL (WONFORD) | Barrack Rd, Exeter, Devon | EX2 5DW |
| Dr Michael Gonzales | Gonzalez | CHARING CROSS HOSPITAL | Fulham Palace Road, London | W6 8RF |
| Dr Thomas D Goode | Goode | POOLE GENERAL HOSPITAL | Longfleet Road, Poole | BH15 2JB |
| Ms E M Gordon | Gordon | ST GEORGE'S HOSPITAL | Blackshaw Road, Tooting, London | SW17 0QT |
| Mr Richard L Gower | Gower | ROYAL GWENT HOSPITAL | Cardiff Road, Newport,Gwent | NP9 2UB |
| Dr John Graham | Graham | SOUTHMEAD HOSPITAL | Westbury-on-Trym, Bristol, Somerset | BS10 5NB |
| Dr Warren Grant | Grant | HEREFORD COUNTY HOSPITAL | Union Walk, Hereford | HR1 2ER |
| Dr Emma Gray | Gray | MUSGROVE PARK HOSPITAL | Mugrove Park, Taunton | TA1 5DA |
| Mr Damian Green | Green | SUNDERLAND ROYAL HOSPITAL | Kayll Road, Sunderland, Tyne and Wear | SR4 7TP |
| Mr Jonathan Greenland | Greenland | STOKE MANDEVILLE HOSPITAL | Mandeville Road, Aylesbury, Buckinghamshire | HP21 8AL |
| Dr Robert Grieve | Grieve | UNIVERSITY HOSPITAL OF COVENTRY AND WARWICKSHIRE | Clifford Bridge Road, Walsgrave, Coventry | CV2 2DX |
| Mr Thomas R L Griffiths | Griffiths | LEICESTER GENERAL HOSPITAL | Gwendolen Road, Leicester | LE5 4PW |
| Mr Sandy Gujral | Gujral | QUEENS HOSPITAL, ROMFORD | Rom Valley Way, Romford, Essex | RM7 0AG |
| Dr Nishi Gupta | Gupta | PRINCESS ALEXANDRA HOSPITAL | Cancer Services, Galen House, Hamstel Road, Harlow, Essex | CM20 1QX |

| | | | | |
|---|---|---|---|---|
| Mr Riza Murat Gurun | Gurun | UNIVERSITY HOSPITAL AYR | Dalmellington Road, Ayr, Scotland | KA6 6DX |
| Mr Peter J Guy | Guy | SALISBURY DISTRICT HOSPITAL | Odstock Road, Salisbury, Wilts | SP2 8BJ |
| Mr Neil Haldar | Haldar | WYCOMBE GENERAL HOSPITAL | Queen Alexander Rd, High Wycombe, Buckinghamshire | HP11 2TT |
| Mr N Halder | Halder | WYCOMBE GENERAL HOSPITAL | Queen Alexander Rd, High Wycombe, Buckinghamshire | HP11 2TT |
| Dr Hall | Hall | ST MARY'S HOSPITAL (ISLE OF WIGHT) | Parkhurst Road , Newport, Isle of Wight | PO30 5TG |
| Professor F C Hamdy | Hamdy | ROYAL HALLAMSHIRE HOSPITAL | Glossop Road, Sheffield | S10 2JF |
| Dr Abdel Hamid | Hamid | BROOMFIELD HOSPITAL | Court Road, Broomfield, Chelmsford, Essex | CM1 5ET |
| Dr C Hamilton | Hamilton | SCARBOROUGH GENERAL HOSPITAL | Woodlands Drive, Scarborough | YO12 6QL |
| Mr John Hammonds | Hammonds | DERRIFORD HOSPITAL | Derriford Road, Plymouth | PL6 8DH |
| Mr S J Hampson | Hampson | QUEEN MARY'S UNIVERSITY HOSPITAL | Roehampton Lane, London | SW15 5PN |
| Mr Damien C Hanbury | Hanbury | LISTER HOSPITAL | Corey’s Mill Lane, Stevenage, Herts | SG1 4AB |
| Mr Ahsanul Haq | Haq | ROYAL PRESTON HOSPITAL | Sharoe Green Lane North, Fulwood, Preston, Lancashire | PR2 9HT |
| Dr P D John Hardman | Hardman | JAMES COOK UNIVERSITY HOSPITAL | Department of Urology, Marton Road, Middlesbrough | TS4 3BW |
| Dr Stephen J Harland | Harland | UNIVERSITY COLLEGE HOSPITAL | 237 Euston Rd, Fitzrovia, London | NW1 2BU |
| Mr John M Harney | Harney | HUDDERSFIELD ROYAL INFIRMARY | Lindley, Huddersfield | HD3 3EA |
| Dr Peter Harper | Harper | GUY'S HOSPITAL | St Thomas Street, London | SE1 9RT |
| Dr Sarah Harris | Harris | PRINCESS ROYAL UNIVERSITY HOSPITAL ORPINGTON | Farnborough Common, Orpington | BR6 8ND |
| Mr D Harris | Harris | ROYAL MARSDEN HOSPITAL | Downs Road, Sutton, Surrey | SM2 5PT |
| Mr G S M Harrison | Harrison | ROYAL HAMPSHIRE COUNTY HOSPITAL | Romsey Road, Winchester, Hants | SO22 5DG |
| Mr D R Harriss | Harriss | NOTTINGHAM CITY HOSPITAL | Hucknall Road, Nottingham | NG5 1PB |
| Mr N Harvey-Hills | Harvey-Hills | PRINCESS MARGARET HOSPITAL | Osborne Road, Windsor, Berks | SL4 3SJ |
| Dr Erik Havranek | Havranek | NORTHWICK PARK HOSPITAL | Watford Road, Harrow | HA1 3UJ |
| Mr Simon Hawkyard | Hawkyard | SCARBOROUGH GENERAL HOSPITAL | Woodlands Drive, Scarborough | YO12 6QL |
| Dr Catherine M Heath | Heath | ROYAL SOUTH HANTS HOSPITAL | The Wessex Rt Centre,St Mary’s Road, Southampton | SO14 0YG |
| Mr Michael Hehir | Hehir | FALKIRK & DISTRICT ROYAL INFIRMARY | Major's Loan, Falkirk, Scotland | FK1 5QE |
| Mr Giles O Hellawell | Hellawell | NORTHWICK PARK HOSPITAL | Watford Road, Harrow | HA1 3UJ |
| Dr R Alexander | Henderson | CUMBERLAND INFIRMARY | Cumberland Infirmary, Newtown Road, Carlisle, Cumbria | CA2 7HY |
| Mr David Hendry | Hendry | BEATSON ONCOLOGY CENTRE | Western Infirmary, Dumbarton Road, Glasgow | G11 6NT |
| Mr Mike Henley | Henley | DERBY CITY GENERAL HOSPITAL | Uttoxeter Road, Derby | DE22 3NE |
| Dr Ann Henry | Henry | BRADFORD ROYAL INFIRMARY | Duckworth Lane, Bradford | BD9 6RJ |
| Dr John Hetherington | Hetherington | CASTLE HILL HOSPITAL | Castle Road, Cottingham, East Yorkshire | HU16 5JQ |
| Dr Tamas Hickish | Hickish | POOLE GENERAL HOSPITAL | Longfleet Road, Poole | BH15 2JB |
| Mr James A Hicks | Hicks | ST RICHARD'S HOSPITAL | Spitalfields Lane, Chichester, West Sussex | PO19 6SE |
| Dr Serena Hilman | Hilman | WESTON GENERAL HOSPITAL | Grange Road, Weston super Mare, North Somerset | BS23 4TQ |
| Mr Mustafa Hilmy | Hilmy | YORK HOSPITAL | Wiggington Road, York, Yorkshire | YO31 8HE |
| Mr Richard Hindley | Hindley | BASINGSTOKE AND NORTH HAMPSHIRE HOSPITAL | Aldermaston Road, Basingstoke, | RG24 9NA |
| Mr John R Hindmarsh | Hindmarsh | JAMES COOK UNIVERSITY HOSPITAL | Department of Urology, Marton Road, Middlesbrough | TS4 3BW |
| Mr John Hines | Hines | WHIPPS CROSS UNIVERSITY HOSPITAL | Whipps Cross Road, Leytonstone, London | E11 1NR |
| Dr M Hingorani | Hingorani | SCARBOROUGH GENERAL HOSPITAL | Woodlands Drive, Scarborough | YO12 6QL |
| Mr Edwin T S Ho | Ho | NORFOLK & NORWICH UNIVERSITY HOSPITAL | Colney Lane, Norwich | NR4 7UZ |
| Professor Shirley Hodgson | Hodgson | ST GEORGE’S HOSPITAL | Blackshaw Road, Tooting, London | SW17 0RE |
| Dr U Hoffman | Hoffman | HUDDERSFIELD ROYAL INFIRMARY | Lindley, Huddersfield | HD3 3EA |
| Mr David Holden | Holden | MACCLESFIELD DISTRICT GENERAL HOSPITAL | Victoria Road, Macclesfield, Cheshire | SK10 3BL |
| Dr A Hollingdale | Hollingdale | PETERBOROUGH CITY HOSPITAL | Edith Cavell Campus, Bretton Gate, Peterborough | PE3 9GZ |
| Mr Graham W Hollins | Hollins | UNIVERSITY HOSPITAL AYR | Dalmellington Road, Ayr, Scotland | KA6 6DX |
| Mr Simon A V Holmes | Holmes | ST MARY'S HOSPITAL. PORTSMOUTH | Milton Road, Portsmouth | PO3 6AD |
| Dr Gail Horan | Horan | QUEEN ELIZABETH HOSPITAL | Mindelsohn Way, Edgbaston, Birmingham | B15 2TH |
| Professor Alan Horwich | Horwich | ROYAL MARSDEN HOSPITAL | Fulham Road, London | SW3 6JJ |
| Professor Peter Hoskin | Hoskin | MOUNT VERNON CENTRE FOR CANCER TREATMENT | Rickmansworth Road, Northwood, Middlesex | HA6 2RN |
| Matthew Hotston | Hotston | ROYAL CORNWALL HOSPITAL (TRELISKE) | Treliske, Truro, Cornwall | TR1 3LJ |
| Dr Joe Houghton | Houghton | BELFAST CITY HOSPITAL | Lisburn Road, Belfast, Northern Ireland | BT9 7AB |
| Dr Houghton | Houghton | CRAIGAVON AREA HOSPITAL | 68 Lurgan Road, Portadown, Craigavon | BT63 5QQ |
| Mr Graham P Howell | Howell | ROYAL UNITED HOSPITAL BATH | Combe Park, Bath | BA1 3NG |
| Mr D Hrouda | Hrouda | CHARING CROSS HOSPITAL | Fulham Palace Road, London | W6 8RF |
| Dr Robert Huddart | Huddart | ROYAL MARSDEN HOSPITAL | Fulham Road, London | SW3 6JJ |
| Ms Liz Hudson | Hudson | ST JAMES'S UNIVERSITY HOSPITAL | Beckett Street, Leeds, West Yorkshire | LS9 7TF |
| Dr Rob Hughes | Hughes | MOUNT VERNON CENTRE FOR CANCER TREATMENT | Rickmansworth Road, Northwood, Middlesex | HA6 2RN |
| Mr Michael Hughes | Hughes | QUEEN ELIZABETH HOSPITAL | Mindelsohn Way, Edgbaston, Birmingham | B15 2TH |
| Mr Owen Hughes | Hughes | UNIVERSITY HOSPITAL OF WALES | Heath Park, Cardiff, Wales | CF14 4XW |
| Dr Abigail Hullingdale | Hullingdale | PETERBOROUGH CITY HOSPITAL | Edith Cavell Campus, Bretton Gate, Peterborough | PE3 9GZ |
| Dr Caroline Humber | Humber | UNIVERSITY HOSPITAL OF COVENTRY AND WARWICKSHIRE | Clifford Bridge Road, Walsgrave, Coventry | CV2 2DX |

| | | | | |
|---|---|---|---|---|
| Mr Jawad Husain | Husain | ROYAL ALBERT EDWARD INFIRMARY | Wigan Lane, Wigan, Lancashire | WN1 2NN |
| Mr Zahid Hussain | Hussain | ROYAL OLDHAM HOSPITAL | Rochdale Road, Oldham, Greater Manchester | OL1 2JH |
| Mr Zahid Hussein | Hussein | NORTH MANCHESTER GENERAL HOSPITAL | Delauneys Road, Crumpsall, Manchester | M8 5RB |
| Mr John W Iacovou | Iacovou | GREAT WESTERN HOSPITAL | Marlborough Road, Swindon, Wilts | SN3 6BB |
| Dr A Ibrahim | Ibrahim | CLATTERBRIDGE CENTRE FOR ONCOLOGY | Clatterbridge Rd, Bebington, Wirral | CH63 4JY |
| Mr P Christian Illie | Illie | THE QUEEN ELIZABETH HOSPITAL | Gayton Road, King's Lynn, Norfolk | PE30 4ET |
| Mr John A Inglis | Inglis | NEW CROSS HOSPITAL | Wednesfield Road, Wolverhampton, West Midlands | WV10 0QP |
| Mr Stuart Irving | Irving | NORFOLK & NORWICH UNIVERSITY HOSPITAL | Colney Lane, Norwich | NR4 7UZ |
| Mr C Irwin | Irwin | ALEXANDRA HOSPITAL | Woodrow Drive, Redditch, Worcestershire | B98 7UB |
| Mr Rami Issa | Issa | ST GEORGE'S HOSPITAL (TOOTING) | Blackshaw Road, Tooting, London | SW17 0QT |
| Dr Louise Izatt | Izatt | GUY'S HOSPITAL | St Thomas Street, London | SE1 9RT |
| Mr Victor Izegbu | Izegbu | CENTRAL MIDDLESEX HOSPITAL | Acton Lane, Park Royal, London | NW10 7NS |
| Mr Ramasamy Jaganathan | Jaganathan | GOOD HOPE HOSPITAL | Rectory Road, Sutton Coldfield, West Midlands | B75 7RR |
| Dr Satinder Jagdev | Jagdev | LEEDS GENERAL INFIRMARY | Great George Street, Leeds, West Yorkshire | LS1 3EX |
| Dr Suneil Jain | Jain | BELFAST CITY HOSPITAL | Lisburn Road, Belfast, Northern Ireland | BT9 7AB |
| Mr Sunjay Jain | Jain | LEEDS GENERAL INFIRMARY | Great George Street, Leeds, West Yorkshire | LS1 3EX |
| Mr Arun Jain | Jain | ROYAL OLDHAM HOSPITAL | Rochdale Road, Oldham, Greater Manchester | OL1 2JH |
| Dr Jalil | Jalil | NORTHWICK PARK HOSPITAL | Watford Road, Harrow | HA1 3UJ |
| Mr Basharat Jameel | Jameel | BRONGLAIS GENERAL HOSPITAL | Caradog Road, Aberystwyth | SY23 1ER |
| Mr Michael J James | James | CHESTERFIELD ROYAL HOSPITAL | Calow, Chesterfield, Derbyshire | S44 5BL |
| Professor N James | James | QUEEN ELIZABETH HOSPITAL | Mindelsohn Way, Edgbaston, Birmingham | B15 2TH |
| Mr R Lester James | James | STAFFORDSHIRE GENERAL HOSPITAL | Weston Road, Stafford, Staffordshire | ST16 3SA |
| Mr Khalid Janjua | Janjua | QUEEN MARGARET HOSPITAL | Whitefield Road, Dunfermline | KY12 0SU |
| Mr Pradip Javle | Javle | LEIGHTON HOSPITAL | Middlewich Road, Crewe, Cheshire | CW1 4QT |
| Kieren Jefferson | Jefferson | UNIVERSITY HOSPITAL (COVENTRY) | Clifford Bridge Road, Walsgrave, Coventry | CV2 2DX |
| Dr Lucy Jellett | Jellett | ALTNAGELVIN AREA HOSPITAL | Glennshane Road, Londonderry | BT47 6SB |
| Dr P Jenkins | Jenkins | CHELTENHAM GENERAL HOSPITAL | Sandford Road, Cheltenham, Gloucester | GL53 7AN |
| Dr Anil Jha | Jha | HEREFORD COUNTY HOSPITAL | Union Walk, Hereford | HR1 2ER |
| Dr Sameer Jhavar | Jhavar | ROYAL MARSDEN HOSPITAL | Fulham Road, London | SW3 6JJ |
| Mr John | John | ST GEORGES UNIVERSITY HOSPITAL | Blackshaw Road, Tooting, London | SW17 0QT |
| Dr Ruth | Johnston | BELFAST CITY HOSPITAL | Lisburn Road, Belfast, Antrim | BT9 7AB |
| Dr Gareth Jones | Jones | BEATSON ONCOLOGY CENTRE | Western Infirmary, Dumbarton Road, Glasgow | G11 6NT |
| Mr Chris R Jones | Jones | ST HELIER HOSPITAL | Wrythe Green Lane,Carshalton, Surrey | SM5 1AA |
| Mr David A Jones | Jones | WARWICK HOSPITAL | Lakin Road, Warwick, Warks | CV34 5BW |
| Mr J Joseph | Joseph | HARROGATE AND DISTRICT HOSPITAL | Lancaster Park Road, Harrogate | HG2 7SX |
| Dr Shelagh Joss | Joss | ST JAMES' UNIVERSITY HOSPITAL | Beckett Street, Leeds, West Yorkshire | LS9 7TF |
| Mr Kabir | Kabir | ST GEORGE'S HOSPITAL (TOOTING) | Blackshaw Road, Tooting, London | SW17 0QT |
| Dr Pawel Kazmarek | Kaczmarek | EAST SURREY HOSPITAL | Canada Ave, Redhill, Surrey | RH1 5RH |
| Mr Amir Kaisary | Kaisary | ROYAL FREE HOSPITAL | Pond Street, London | NW3 2QG |
| Dr Alexandre L Kaliski | Kaliski | ST BARTHOLOMEW'S HOSPITAL | West Smithfield, London | EC1A 7BE |
| Dr Jasjit Kalsi | Kalsi | CHARING CROSS HOSPITAL | Fulham Palace Road, London | W6 8RF |
| Dr G Kapur | Kapur | NORFOLK AND NORWICH UNIVERSITY HOSPITAL | Colney Lane, Norwich | NR4 7UY |
| Mr O Karim | Karim | WEXHAM PARK HOSPITAL | Wexham street, Slough, Berkshire | SL2 4HL |
| Dr Stephen J Karp | Karp | CHASE FARM HOSPITAL | The Ridgeway, Enfield, Middlesex | EN2 8JL |
| Mr F X Keeley | Keeley | SOUTHMEAD HOSPITAL | Westbury-on-Trym, Bristol, Somerset | BS10 5NB |
| Dr Penny Kehagioglou | Kehagioglou | ROYAL UNITED HOSPITAL BATH | Combe Park, Bath | BA1 3NG |
| Mr Anand R Kelkar | Kelkar | HAMMERSMITH HOSPITAL | Du Cane Road, London | W12 0HS |
| Mr J P Kelleher | Kelleher | WYCOMBE GENERAL HOSPITAL | Queen Alexander Rd, High Wycombe, Buckinghamshire | HP11 2TT |
| Mr John Kelly | Kelly | UNIVERSITY COLLEGE HOSPITAL LONDON | 243 Euston Rd, Fitzrovia, London | NW1 2BU |
| Dr Sue Kenwrick | Kenwrick | ADDENBROOKE'S HOSPITAL | Hills Road, Cambridge | CB2 2QQ |
| Dr Sara Khaksar | Khaksar | ASHFORD HOSPITAL | London Road, Stanwell, Ashford | TW15 3AA |
| Mr F Khan | Khan | EDITH CAVELL HOSPITAL | Bretton Gate, Peterborough, Cambridgeshire | PE3 9GZ |
| Mr. Altaf Q. Khattak | Khattak | ST HELENS HOSPITAL | Marshalls Cross Road, St Helens | WA9 3DA |
| Dr Vincent Khoo | Khoo | ROYAL MARSDEN HOSPITAL | Fulham Road, London | SW3 6JJ |
| Mr Bijan Khoubehi | Khoubehi | CHELSEA & WESTMINSTER HOSPITAL | 369 Fulham Road, London | SW10 9NH |
| Mr Sikandar Khwaja | Khwaja | QUEEN'S HOSPITAL BURTON UPON TRENT | Belvedere Road, Burton-On-Trent, Derbyshire | DE13 0RB |
| Dr Ann Kiltie | Kiltie | CHURCHILL HOSPITAL | Old Road, Headington, Oxford | OX3 7LE |
| Ms Rachel M Kimber | Kimber | HARROGATE DISTRICT HOSPITAL | Lancaster Park Road, Harrogate | HG2 7SX |
| Mr R Kinder | Kinder | CHELTENHAM GENERAL HOSPITAL | Sandford Road, Cheltenham, Gloucester | GL53 7AN |
| Professor Roger S Kirby | Kirby | THE PROSTATE CENTRE | 32 Wimpole Street, London | W1G 8GT |
| Professor David Kirk | Kirk | BEATSON ONCOLOGY CENTRE | Western Infirmary, Dumbarton Road, Glasgow | G11 6NT |

| | | | | |
|---|---|---|---|---|
| Dr Peter Kirkbride | Kirkbride | WESTON PARK HOSPITAL | Whitham Road, Sheffield, South Yorkshire | S10 2SJ |
| Mr Magdi M Kirollos | Kirollos | TORBAY HOSPITAL | Lawes Bridge, Torquay, Devon | TQ2 7AA |
| Mr Ross Knight | Knight | YSBYTY GLAN CLWYD | Rhuddlan Rd, Bodelwyddan, Rhyl | LL18 5UJ |
| Mr Roger Kockelbergh | Kockelbergh | LEICESTER GENERAL HOSPITAL | Gwendolen Road, Leicester | LE67 4DE |
| Mr Philip C W C Koenig | Koenig | AIREDALE GENERAL HOSPITAL | Skipton Road, Steeton, Keighley, West Yorkshire | BD20 6TD |
| Dr Koh | Koh | KIDDERMINSTER HOSPITAL | Bewdley Road , Kidderminster, Worcestershire | DY11 6RJ |
| Mr S. Koneru | Koneru | MANOR HOSPITAL | Moat Road, Walsall, West Midlands | WS2 9PS |
| Mr Vincent Koo | Koo | KIDDERMINSTER HOSPITAL | Bewdley Road , Kidderminster, Worcestershire | DY11 6RJ |
| Mr Gordon G Kooiman | Kooiman | DARENT VALLEY HOSPITAL | Darenth Wood Road, Darford, Kent | DA2 8DA |
| Dr O Koreich | Koreich | SOUTHEND UNIVERSITY HOSPITAL | Prittlewell Chase, Westcliff on Sea, Essex | SS0 0RY |
| Mr Anthony Koupparis | Koupparis | SOUTHMEAD HOSPITAL | Westbury-on-Trym, Bristol, Somerset | BS10 5NB |
| Mr Mohamed Kourah | Kourah | ROCHDALE INFIRMARY | Whitehall Street, Rochdale, Greater Manchester | OL12 0NB |
| Dr Sigurd Kraus | Kraus | CASTLE HILL HOSPITAL | Castle Road, Cottingham, East Yorkshire | HU16 5JQ |
| Ms Magda L Kujawa | Kujawa | STEPPING HILL HOSPITAL | Poplar Grove, Hazel Grove, Stockport | SK2 7JE |
| Mr Ravi Kulkarni | Kulkarni | ASHFORD HOSPITAL | London Road, Stanwell, Ashford | TW15 3AA |
| Mr M Kumar | Kumar | QUEENS HOSPITAL BURTON | Belvedere Road, Burton-On-Trent, Derbyshire | DE13 0RB |
| Dr Ian H Kunkler | Kunkler | DUMFRIES & GALLOWAY ROYAL INFIRMARY | Bankend Road, Dumfries | DG1 4AP |
| Dr Kurec | Kurec | ALEXANDRA HOSPITAL | Woodrow Drive, Redditch, Worcestershire | B98 7UB |
| Mr Paul Kutarski | Kutarski | ARROWE PARK HOSPITAL | Arrowe Park Road, Upton , Wirral, Merseyside | CH49 5PE |
| Professor H Kynaston | Kynaston | UNIVERSITY HOSPITAL OF WALES | Heath Park, Cardiff, Wales | CF14 4XW |
| Dr Katherine L Lachlan | Lachlan | PRINCESS ANNE HOSPITAL | Coxford Rd, Southampton, Hants | SO16 5YA |
| Dr Robert Laing | Laing | ROYAL SURREY COUNTY HOSPITAL | Egerton Road, Guildford | GU2 5XX |
| Dr Fiona Lalloo | Lalloo | CHRISTIE HOSPITAL | Wilmslow Road, Withington, Manchester | M20 4QL |
| Dr Carolyn Lamb | Lamb | BEATSON WEST OF SCOTLAND CANCER CENTRE | Western Infirmary, Dumbarton Road, Glasgow | G11 6NT |
| Mr M Lancashire | Lancashire | ALEXANDRA HOSPITAL | Woodrow Drive, Redditch, Worcestershire | B98 7UP |
| Mr Stephen E M Langley | Langley | FRIMLEY PARK HOSPITAL | Portsmouth Road, Frimley, Camberley | GU16 7UJ |
| Mr Marc Laniado | Laniado | WEXHAM PARK HOSPITAL | Wexham street, Slough, Berkshire | SL2 4HL |
| Mr T R Larner | Larner | ROYAL SUSSEX COUNTY HOSPITAL | Eastern Road, Brighton, East Sussex | BN2 5BE |
| Mr Latif | Latif | WREXHAM MAELOR HOSPITAL | Croesnewydd Road, Wrexham | LL13 7TD |
| Mr Maurice W Lau | Lau | ROYAL ALBERT EDWARD INFIRMARY | Wigan Lane, Wigan, Lancashire | WN1 2NN |
| Mr W T Lawrence | Lawrence | EASTBOURNE DISTRICT GENERAL HOSPITAL | King's Drive, Eastbourne, East Sussex | BN21 2UD |
| Miss Anne Lawson | Lawson | HARROGATE DISTRICT HOSPITAL | Lancaster Park Road, Harrogate | HG2 7SX |
| Mr Pieter J Le Roux | Le Roux | EPSOM GENERAL HOSPITAL | Dorking Road, Epsom, Surrey | KT18 7EG |
| Professor Mary Leader | Leader | BEAUMONT HOSPITAL | Beaumont Road, Dublin, Southern Ireland | Dublin 9 |
| Mr J O Lee | Lee | NOBLE'S HOSPITAL | Westmorland Road, Douglas, Isle of Man | IM1 4QA |
| Ms L Lee | Lee | ROYAL BOLTON HOSPITAL | Minerva Road, Farnworth, Bolton, Lancs | BL4 0JR |
| Ms A Lee | Lee | ST BARTHOLOMEW’S HOSPITAL | West Smithfield, London | EC1A 7BE |
| Dr Kathryn Lees | Lees | THE MAIDSTONE HOSPITAL | Hermitage Lane, Maidstone, Kent | ME16 9QQ |
| Dr R John Lemburger | Lemburger | NOTTINGHAM CITY HOSPITAL | Hucknall Road, Nottingham | NG5 1PB |
| Dr Priscilla Leone | Leone | BROOMFIELD HOSPITAL | Court Road, Broomfield, Chelmsford, Essex | CM1 5ET |
| Mr Thomas Leslie | Leslie | MILTON KEYNES HOSPITAL | Standing  Way, Eaglestone, Milton Keynes | MK6 5LD |
| Dr Jason Lester | Lester | VELINDRE HOSPITAL | Whitchurch, Cardiff, Wales | CF14 2TL |
| Mr Hing Leung | Leung | NEWCASTLE GENERAL HOSPITAL | Westgate Road, Newcastle-upon-Tyne | NE4 6BE |
| Mr John Leveckis | Leveckis | DONCASTER ROYAL INFIRMARY (BASSETLAW HOSPITAL) | Armthorpe Road, Doncaster | DN2 5LT |
| Mr J Lewis | Lewis | MAIDSTONE HOSPITAL | Hermitage Lane, Barming, Kent | ME16 9QQ |
| Mr D Christopher Lewis | Lewis | WARWICK HOSPITAL | Lakin Road, Warwick, Warks | CV34 5BW |
| Dr Mark Linch | Linch | UNIVERSITY COLLEGE HOSPITAL | 240 Euston Rd, Fitzrovia, London | NW1 2BU |
| Mr Thomas Liston | Liston | WORTHING HOSPITAL | Lyndhurst Road, Worthing, West Sussex | BN11 2DH |
| Dr John Littler | Littler | THE CLATTERBRIDGE CANCER CENTRE | Clatterbridge Rd,  Bebington, Wirral | CH63 4JY |
| Dr John Littler | Littler | THE CLATTERBRIDGE CANCER CENTRE | Clatterbridge Rd,  Bebington, Wirral | CH63 4JY |
| Dr Jacqueline Livsey | Livsey | CHRISTIE HOSPITAL | Wilmslow Road, Withington, Manchester | M20 4BX |
| Mr S Lloyd | Lloyd | STIRLING ROYAL INFIRMARY | Livilands Gate, Stirling | FK8 2AU |
| Dr Imogen Locke | Locke | ROYAL MARSDEN HOSPITAL | Fulham Road, London | SW3 6JJ |
| Mr Richard Lodge | Lodge | SOUTHEND UNIVERSITY HOSPITAL | Prittlewell Chase, Westcliff on Sea, Essex | SS0 0RY |
| Dr John Logue | Logue | CHRISTIE HOSPITAL | Wilmslow Road, Withington, Manchester | M20 4BX |
| Mr Mark Longmuir | Longmuir | W SCOTLAND REGIONAL GENETICS SERVICE | Yorkhill, Glasgow | G3 8SJ |
| Dr Carmel Loughrey | Loughrey | LEEDS GENERAL INFIRMARY | Great George Street, Leeds, West Yorkshire | LS1 3EX |
| Mr Malcolm G Lucas | Lucas | MORRISTON HOSPITAL | Morriston, Swansea, Wales | SA6 6NL |
| Mr C J Luscombe | Luscombe | QUEEN ELIZABETH HOSPITAL | Mindelsohn Way, Edgbaston, Birmingham | B15 2TH |
| Dr Anna Lydon | Lydon | TORBAY HOSPITAL | Lawes Bridge, Torquay, Devon | TQ2 7AA |
| Mr Michael Lynch | Lynch | COLCHESTER GENERAL HOSPITAL | Turner Road, Colchester, Essex | CO4 5JL |

| | | | | |
|---|---|---|---|---|
| Mr Naing N K Lynn | Lynn | ROYAL SHREWSBURY HOSPITAL | Mytton Oak Road, Shrewsbury | SY3 8XQ |
| Mr James P A MacDermott | MacDermott | TORBAY HOSPITAL | Lawes Bridge, Torquay, Devon | TQ2 7AA |
| Mr Ruaraidh P Macdonagh | Macdonagh | TAUNTON AND SOMERSET HOSPITAL | Musgrove Park, Taunton, Somerset | TA1 5DA |
| Mr Macdonald | Macdonald | UNIVERSITY HOSPITAL COVENTRY AND WARWICKSHIRE | Clifford Bridge Road, Walsgrave, Coventry | CV2 2DX |
| Mr Sanjeev Madaan | Madaan | DARENT VALLEY HOSPITAL | Darenth Wood Road, Darford, Kent | DA2 8DA |
| Mr Satish Maddineni | Maddineni | SALFORD ROYAL | Stott Lane, Salford | M6 8HD |
| Dr Kudingila R Madhava | Madhava | ST MARY'S HOSPITAL. PORTSMOUTH | Milton Road, Portsmouth | PO3 6AD |
| Mr Vijay Madhukar | Madhukar | UNIVERSITY HOSPITAL OF NORTH TEES | Hardwick, Stockton on Tees | TS19 8PE |
| Dr Joseph Maguire | Maguire | CLATTERBRIDGE CENTRE FOR ONCOLOGY | Clatterbridge Rd, Bebington, Wirral | CH63 4JY |
| Professor E R Maher | Maher | BIRMINGHAM WOMEN'S HOSPITAL | Clinical Genetics Unit, Edgbaston, Birmingham | B15 2TG |
| Mr Wasim Mahmalji | Mahmalji | HEREFORD COUNTY HOSPITAL | Stonebow Road , Hereford, Herefordshire | HR1 2BN |
| Dr Rana Mahmood | Mahmood | UNIVERSITY HOSPITAL AYR | Dalmellington Road, Ayr, Scotland | KA6 6DX |
| Dr Graeme H M Mair | Mair | ST BARTHOLOMEWS HOSPITAL | West Smithfield, London | EC1A 7BE |
| Mr Adel Makar | Makar | ALEXANDRA HOSPITAL | Woodrow Drive, Redditch, Worcestershire | B98 7UB |
| Mr John Makunde | Makunde | ST MARY'S HOSPITAL (ISLE OF WIGHT) | Parkhurst Road , Newport, Isle of Wight | PO30 5TG |
| Dr Zafar Malik | Malik | QUEEN MARGARET HOSPITAL | Whitefield Road, Dunfermline | KY12 0SU |
| Mr Peter R Malone | Malone | BATTLE HOSPITAL | Oxford Road, Reading, Berkshire | RG3 1AG |
| Dr Caroline Manetta | Manetta | EASTBOURNE DISTRICT GENERAL HOSPITAL | King's Drive, Eastbourne, East Sussex | BN21 2UD |
| Dr Stephen A Mangar | Mangar | HAMMERSMITH HOSPITAL | Du Cane Road, London | W12 0HS |
| Mr Manikandan | Manikandan | ROYAL ALBERT EDWARD INFIRMARY | Wigan Lane, Wigan, Lancashire | WN1 2NN |
| Mr Mark Mantle | Mantle | ROYAL UNITED HOSPITAL BATH | Combe Park, Bath | BA1 3NG |
| Mr I Mark | Mark | LINCOLN COUNTY HOSPITAL | Greetwell Road, Lincoln | LN2 5QY |
| Mr Howard Marsh | Marsh | MEDWAY MARITIME HOSPITAL | Windmill Road, Gillingham | ME7 5NY |
| Dr Alex Martin | Martin | HINCHINGBROOKE HOSPITAL | Hinchingbrooke Park , Huntingdon, Cambridgeshire | PE29 6NT |
| Mr Robert Mason | Mason | TORBAY HOSPITAL | Lawes Bridge, Torquay, Devon | TQ2 7AA |
| Professor M D Mason | Mason | VELINDRE HOSPITAL | Whitchurch, Cardiff, Wales | CF14 2TL |
| Mr Shikohe Masood | Masood | MEDWAY MARITIME HOSPITAL | Windmill Road, Gillingham | ME7 5NY |
| Mr Matanhelia | Matanhelia | CHORLEY AND SOUTH RIBBLE DISTRICT GENERAL HOSPITAL | Preston Road, Chorley, Lancashire | PR7 1PP |
| Mr Shyam Matenhelia | Matenhelia | ROYAL PRESTON HOSPITAL | Sharoe Green Lane North, Fulwood, Preston, Lancashire | PR2 9HT |
| Mr Philip N Matthews | Matthews | UNIVERSITY HOSPITAL OF WALES | Heath Park, Cardiff, Wales | CF14 4XW |
| Mr Erik Mayer | Mayer | ST MARY'S HOSPITAL, LONDON | Praed Street, Paddington, London | W2 1NY |
| Dr J McAleese | McAleese | CRAIGAVON AREA HOSPITAL | 68 Lurgan Road, Portadown, Craigavon | BT63 5QQ |
| Ms Donna McBride | McBride | ROYAL BOURNEMOUTH HOSPITAL | Castle Lane East, Bournemouth, Dorset | BH7 7DW |
| Mr. John E. McCabe | McCabe | ST HELENS HOSPITAL | Marshalls Cross Road, St Helens | WA9 3DA |
| Donald MacDonald | McDonald | UNIVERSITY HOSPITAL (COVENTRY) | Clifford Bridge Road, Walsgrave, Coventry | CV2 2DX |
| Mr Jonathan McFarlane | McFarlane | ROYAL UNITED HOSPITAL BATH | Combe Park, Bath | BA1 3NG |
| Dr Adam McGeoch | McGeogh | HINCHINGBROOKE HOSPITAL | Hinchingbrooke Park, Hinchingbrooke, Huntingdon | PE29 6NT |
| Dr Ursula McGovern | McGovern | BARNET HOSPITAL | Wellhouse Lane, Barnet, Herts | EN5 3DJ |
| Dr Ursula McGovern | McGovern | UNIVERSITY COLLEGE HOSPITAL | 241 Euston Rd, Fitzrovia, London | NW1 2BU |
| Dr John McGrane | McGrane | ROYAL CORNWALL HOSPITAL (TRELISKE) | Treliske, Truro, Cornwall | TR1 3LJ |
| Mr McGrath | McGrath | SOUTHMEAD HOSPITAL | Westbury-on-Trym, Bristol, Somerset | BS10 5NB |
| Mr Craig McIlhenny | McIlhenny | FORTH VALLEY ROYAL HOSPITAL | Stirling Road, Larbert | FK5 4WR |
| Mr Paul McInerney | McInerney | DERRIFORD HOSPITAL | Derriford Road, Plymouth | PL6 8DH |
| Mr Gregor McIntosh | McIntosh | SALISBURY DISTRICT HOSPITAL | Odstock Road, Salisbury, Wilts | SP2 8BJ |
| Dr F McKinna | McKinna | EASTBOURNE DISTRICT GENERAL HOSPITAL | Eastern Road, Brighton, East Sussex | BN2 5BE |
| Dr Duncan McLaren | McLaren | WESTERN GENERAL HOSPTIAL | Crewe Road, Edinburgh | EH4 2UX |
| Miss Esther McLarty | McLarty | DERRIFORD HOSPITAL | Derriford Road, Plymouth | PL6 8DH |
| Dr Nicholas McLeod | McLeod | UNIVERSITY HOSPITAL AYR | Dalmellington Road, Ayr, Scotland | KA6 6DX |
| Dr Rhona McMenemin | McMenemin | NEWCASTLE GENERAL HOSPITAL | Westgate Road, Newcastle-upon-Tyne | NE4 6BE |
| Mr Alan McNeill | McNeill | WESTERN GENERAL HOSPTIAL | Crewe Road, Edinburgh | EH4 2UX |
| Mr T A McNicholas | McNicholas | LISTER HOSPITAL | Corey's Mill Lane, Stevenage, Herts | SG1 4AB |
| Mr Robert N Meddings | Meddings | UNIVERSITY HOSPITAL AYR | Dalmellington Road, Ayr, Scotland | KA6 6DX |
| Mr A David Mee | Mee | NORTHWICK PARK HOSPITAL | Watford Road, Harrow | HA1 3UJ |
| Dr Lucinda Melcher | Melcher | PRINCESS ALEXANDRA HOSPITAL | Cancer Services, Galen House, Hamstel Road, Harlow, Essex | CM20 1QX |
| Mr Memon | Memon | PILGRIM HOSPITAL | Sibsey Road, Boston, Lincs | PE21 9QS |
| Mr Pravin Menzes | Menzes | PRINCESS ROYAL HOSPITAL | Lewes Road, Haywards Heath, West Sussex | RH16 4EX |
| Dr J Michels | Michels | TORBAY HOSPITAL | Lowes Bridge, Torquay, Devon | TQ2 7AA |
| Mr Marek Miller | Miller | NORTHAMPTON GENERAL HOSPITAL | Billing Rd, Northampton | NN1 5BD |
| Mr Robert Mills | Mills | NORFOLK & NORWICH UNIVERSITY HOSPITAL | Colney Lane, Norwich | NR4 7UZ |
| Mr Soumya Misra | Misra | NORTH DEVON DISTRICT HOSPITAL | Raleigh Park, Barnstaple, Devon | EX31 4JB |
| Mr. Rahul Mistry | Mistry | ST HELENS HOSPITAL | Marshalls Cross Road, St Helens | WA9 3DA |

| | | | | |
|---|---|---|---|---|
| Mr S Mitchell | Mitchell | WATFORD GENERAL HOSPITAL | Vicarage Road, Watford, Hertfordshire | WD18 0HB |
| Dr Natasha Mithal | Mithal | KENT & CANTERBURY HOSPITAL | Ethelbert Road, Canterbury, Kent | CT1 3NG |
| Dr Anita Mitra | Mitra | ROYAL MARSDEN HOSPITAL | Fulham Road, London | SW3 6JJ |
| Ms Gillian E Mobb | Mobb | ROYAL BOLTON HOSPITAL | Minerva Road, Farnworth, Bolton, Lancs | BL4 0JR |
| Mr Leslie E F Moffat | Moffat | ABERDEEN ROYAL INFIRMARY | Foresterhill, Aberdeen | AB25 22N |
| Mr Mokete | Mokete | ROYAL PRESTON HOSPITAL | Sharoe Green Lane North, Fulwood, Preston, Lancashire | PR2 9HT |
| Mr Jaswant Mom | Mom | WEST CUMBERLAND HOSPITAL | Homewood, Hensingham, Whitehaven | CA28 8JG |
| Dr Julian Money-Kyrle | Money-Kyrle | EAST SURREY HOSPITAL | Canada Ave, Redhill, Surrey | RH1 5RH |
| Mr Richard Mongtague | Montague | WITHINGTON HOSPITAL | Nell Lane, Whitington, Manchester | M20 2LR |
| Mr Bruce Montgomery | Montgomery | FRIMLEY PARK HOSPITAL | Portsmouth Road, Frimley, Camberley | GU16 5UJ |
| Mr Martin P Moody | Moody | NORTH DEVON DISTRICT HOSPITAL | Raleigh Park, Barnstaple, Devon | EX31 4JB |
| Dr Moosa | Moosa | WITHYBUSH GENERAL HOSPITAL | Ward 10, Fishguard Road, Haverford West, Pembrokeshire | SA61 2PZ |
| Mr Roland Morley | Morley | KINGSTON HOSPITAL | Galsworthy Road, Kingston-upon-Thames, Surrey | KT2 7QB |
| Mr Sean B Morris | Morris | SOLIHULL HOSPITAL | Lode Lane, Solihull, West Midlands | B91 2JL |
| Professor Patrick Morrison | Morrison | BELFAST CITY HOSPITAL | Lisburn Road, Belfast, Northern Ireland | BT9 7AB |
| Dr Diana Mort | Mort | PRINCESS OF WALES HOSPITAL | Coity Road, Bridgend | CF31 1RQ |
| Mr Amir H Mostafid | Mostafid | THE HAMPSHIRE CLINIC | Basing Road, Old Basing, Basingstoke | RG24 7AL |
| Mr Hanif Motiwala | Motiwala | KING EDWARD VII HOSPITAL | Windsor, Berkshire | SL4 3DP |
| Mr Gulzar Mufti | Mufti | MEDWAY MARITIME HOSPITAL | Windmill Road, Gillingham | ME7 5NY |
| Mr Gordon Muir | Muir | KING'S COLLEGE HOSPITAL | Denmark Hill, London | SE5 9RS |
| Dr Colin Mulholland | Mulholland | ATNAGELVIN AREA HOSPITAL | Londonderry, Northern Ireland | NT47 6SB |
| Mr Faiz Mumtaz | Mumtaz | BARNET GENERAL HOSPITAL | Wellhouse Lane, Barnet, Herts | EN5 3DJ |
| Nicholas Munro | Munro | ROYAL CORNWALL HOSPITAL (TRELISKE) | Treliske, Truro, Cornwall | TR1 3LJ |
| Dr Murad | Murad | CUMBERLAND INFIRMARY | Cumberland Infirmary, Newtown Road, Carlisle, Cumbria | CA2 7HY |
| Mr Michael Murphy | Murphy | PINDERFIELDS HOSPITAL | Aberford Road, Wakefield, West Yorkshire | WF5 4DG |
| Mr Keith W Murray | Murray | KENT & CANTERBURY HOSPITAL | Ethelbert Road, Canterbury, Kent | CT1 3NG |
| Dr Alexandra Murray | Murray | UNIVERSITY HOSPITAL OF WALES | Heath Park, Cardiff, Wales | CF14 4XW |
| Dr Shirley Murrell | Murrell | ROYAL SUSSEX COUNTY HOSPITAL | Eastern Road, Brighton, East Sussex | BN2 5BE |
| Dr D Muthukumar | Muthukumar | DERBYSHIRE ROYAL INFIRMARY | London Road, Derby | DE1 2QY |
| Mr Andrew Myatt | Myatt | CASTLE HILL HOSPITAL | Castle Road, Cottingham, East Yorkshire | HU16 5JQ |
| Professor Ghulam Nabi | Nabi | NINEWELLS HOSPITAL | Ninewells Avenue, Dundee | DD1 9SY |
| Mr Harry Naerger | Naerger | FRIMLEY PARK HOSPITAL | Portsmouth Road, Frimley, Camberley | GU16 5UJ |
| Dr Yoodhvir Sing Nagar | Nagar | QUEEN ALEXANDRA HOSPITAL | Southwick Hill Road, Cosham, Porthmouth, Hampshire | PO6 3LY |
| Muthuswamy Nagarajan | Nagarajan | ROYAL ALBERT EDWARD INFIRMARY | Wigan Lane, Wigan, Lancashire | WN1 2NN |
| Mr Manu Nair | Nair | HEARTLANDS HOSPITAL | Bordesley Green East, Birmingham | B9 5SS |
| Mr Siva Namasivayam | Namasivayam | MACCLESFIELD DISTRICT GENERAL HOSPITAL | Victoria Road, Macclesfield, Cheshire | SK10 3BL |
| Mr Thiagarajan Nambirajan | Nambirajan | ARROWE PARK HOSPITAL | Arrowe Park Road, Upton , Wirral, Merseyside | CH49 5PE |
| Mr Krishna Narahari | Narahari | UNIVERSITY HOSPITAL OF WALES | Heath Park, Cardiff, Wales | CF14 4XW |
| Mr Vinod Nargund | Nargund | ST BARTHOLOMEWS HOSPITAL | West Smithfield, London | EC1A 7BE |
| Mr Nawrocki | Nawrocki | ROYAL SUSSEX COUNTY HOSPITAL | Eastern Road, Brighton, East Sussex | BN2 5BE |
| Mr Richard Nayar | Nayar | HEREFORD COUNTY HOSPITAL | Union Walk, Hereford | HR1 2ER |
| Professor David Neal | Neal | ADDENBROOKE'S HOSPITAL | Cambridge Biomedical Campus, Hill Road, Cambridge | CB2 0QQ |
| Mr Donald Neilson | Neilson | BLACKBURN ROYAL INFIRMARY | Haslingden Road, Blackburn | BB2 3HH |
| Dr A Nethersell | Nethersell | GLAN CLWYD HOSPITAL | Bodelwyddan, Rhyl, Wales | LL18 5UJ |
| Mr Julian Barwell | NEW | LEICESTER ROYAL INFIRMARY | Infirmary Square, Leicester | LE1 5WW |
| Dr Jacqueline C Newby | Newby | NORTH MIDDLESEX HOSPITAL | Sterling Way, London | N18 1QX |
| Dr Hugh Newman | Newman | ROYAL UNITED HOSPITAL BATH | Combe Park, Bath | BA1 3NG |
| Dr R Newton | Newton | ROYAL CORNWALL HOSPITAL | Treliske, Truro, Cornwall | TR1 3LJ |
| Dr Ashok Damanta Nikapota | Nikapota | WORTHING HOSPITAL | Lyndhurst Road, Worthing, West Sussex | BN11 2DH |
| Dr Jenny Nobes | Nobes | NORFOLK & NORWICH UNIVERSITY HOSPITAL | Colney Lane, Norwich | NR4 7UY |
| Mr Martin Nuttall | Nuttall | BROOMFIELD HOSPITAL | Court Road, Broomfield, Chelmsford, Essex | CM1 5ET |
| Mr Neil Oakley | Oakley | STEPPING HILL HOSPITAL | Poplar Grove, Hazel Grove, Stockport | SK2 7JE |
| Mr Jeremy Oates | Oates | LEIGHTON HOSPITAL | Middlewich Road, Crewe, Cheshire | CW1 4QJ |
| Mr P J O'Boyle | O'Boyle | TAUNTON AND SOMERSET HOSPITAL | Musgrove Park, Taunton, Somerset | TA1  5DA |
| Mr J O'Brien | O'Brien | BIRMINGHAM HEARTLANDS HOSPITAL | Bordesley Green East, Birmingham | B9 5SS |
| Mr Tim S O'Brien | O'Brien | GUY'S HOSPITAL | St Thomas Street, London | SE1 9RT |
| Dr H O'Donnell | O'Donnell | WEXHAM PARK HOSPITAL | Wexham street, Slough, Berkshire | SL2 4HL |
| Mr Neil O'Donoghue | O'Donoghue | HARLEY STREET CONSULTING ROOMS | 99 Harley Street, London | W1G 6AQ |
| Mr E O'Donoghue | O'Donoghue | UNIVERSITY COLLEGE HOSPITAL | 238 Euston Rd, Fitzrovia, London | NW1 2BU |
| Mr Chris Ogden | Ogden | ROYAL MARSDEN HOSPITAL | Fulham Road, London | SW3 6JJ |
| Mr Hemant Ohja | Ojha | GOOD HOPE HOSPITAL | Rectory Road, Sutton Coldfield, West Midlands | B75 7RR |

| | | | | |
|---|---|---|---|---|
| Mr Aloysius Okeke | Okeke | CHELTENHAM GENERAL HOSPITAL | Sandford Road, Cheltenham, Gloucester | GL53 7AG |
| Professor Tim Oliver | Oliver | ST BARTHOLOMEWS HOSPITAL | West Smithfield, London | EC1A 7BE |
| Dr Kai Ren Ong | Ong | BIRMINGHAM WOMEN'S HOSPITAL | Clinical Genetics Unit, Edgbaston, Birmingham | B15 2TG |
| Mr Eng K Ong | Ong | NORTH DEVON DISTRICT HOSPITAL | Raleigh Park, Barnstaple, Devon | EX31 4JB |
| Mr Johnathan Ord | Ord | GLOUCESTERSHIRE ROYAL HOSPITAL | Great Western Road, Gloucester, Gloucestershire | GL1 3NN |
| Mr P O'Reilly | O'Reilly | STEPPING HILL HOSPITAL | Poplar Grove, Hazel Grove, Stockport | SK2 7JE |
| Dr J S O'Rourke | O'Rourke | ROYAL CORNWALL HOSPITAL | Treliske, Truro, Cornwall | TR1 3LJ |
| Mr David Osborn | Osborn | LEICESTER GENERAL HOSPITAL | Gwendolen Road, Leicester | LE5 4PW |
| Dr Peter Ostler | Ostler | MOUNT VERNON CENTRE FOR CANCER TREATMENT | Rickmansworth Road, Northwood, Middlesex | HA6 2RN |
| Professor Joe O'Sullivan | O'Sullivan | BELFAST CITY HOSPITAL | Lisburn Road, Belfast, Northern Ireland | BT9 7AB |
| Dr J Owen | Owen | GLOUCESTERSHIRE ROYAL HOSPITAL | Great Western Road, Gloucester, Gloucestershire | GL1 3NN |
| Dr Nachi Palaniappan | Palaniappan | PRINCESS OF WALES HOSPITAL | Coity Road, Bridgend | CF31 1RQ |
| Mr Edward Palfrey | Palfrey | FRIMLEY PARK HOSPITAL | Portsmouth Road, Frimley, Camberley | GU16 7UJ |
| Mr Victor Palit | Palit | UNIVERSITY HOSPITAL OF NORTH TEES | Hardwick, Stockton on Tees | TS19 8PE |
| Dr Miguel Panades | Panades | LINCOLN COUNTY HOSPITAL | Greetwell Road, Lincoln | LN2 5QY |
| Dr Niki Panakis | Panakis | CHURCHILL HOSPITAL | Old Road, Headington, Oxford | OX3 7LJ |
| Mr M Pancharatnam | Pancharatnam | HEMEL HEMPSTEAD GENERAL HOSPITAL | Hillfield Road, Hemel Hempstead, Herts | HP2 4AD |
| Mr Michalakis L Pantelides | Pantelides | ROYAL BOLTON HOSPITAL | Minerva Road, Farnworth, Bolton, Lancs | BL4 0JR |
| Dr U Panwar | Panwar | BROOMFIELD HOSPITAL | Court Road, Broomfield, Chelmsford, Essex | CM1 5ET |
| Dr Omi Parikh | Parikh | BURNLEY GENERAL HOSPITAL | Casterton Avenue, Burnley, Lancs | BB10 2PQ |
| Dr Chris Parker | Parker | ROYAL MARSDEN HOSPITAL | Fulham Road, London | SW3 6JJ |
| Mr Christopher H Parker | Parker | YEOVIL DISTRICT HOSPITAL | Higher Kingston, Yeovil, Somerset | BA21 4AT |
| Mr Nigel Parr | Parr | ARROWE PARK HOSPITAL | Arrowe Park Road, Upton , Wirral, Merseyside | CH49 5PE |
| Mr Bohdan T Parys | Parys | ROTHERHAM GENERAL HOSPITAL | Moorgate Road, Rotherham | S60 2UD |
| Dr Sarah Pascoe | Pascoe | DERRIFORD HOSPITAL | Derriford Road, Plymouth | PL6 8DH |
| Mr Anup Patel | Patel | ST MARY'S HOSPITAL, LONDON | Praed Street, Paddington, London | W2 1NY |
| Dr Joan Paterson | Paterson | ADDENBROOKE'S HOSPITAL | Hills Road, Cambridge | CB2 2QQ |
| Mr S Pathack | Pathack | DONCASTER ROYAL INFIRMARY | Doncaster Royal Infirmary, C Block, Doncaster Royal Infirmary, Armthorpe Road, Doncaster | DN2 5LT |
| Ms Jhumur Pati | Pati | HOMERTON UNIVERSITY HOSPITAL | Homerton Row, London | E9 6SR |
| Dr Helen Patterson | Patterson | ADDENBROOKE'S HOSPITAL | Hills Road, Cambridge | CB2 2QQ |
| Dr Pattu | Pattu | QUEENS HOSPITAL BURTON | Belvedere Road, Burton-On-Trent, Derbyshire | DE13 0RB |
| Mr A Paul | Paul | ST JAMES' UNIVERSITY HOSPITAL | Beckett Street, Leeds, West Yorkshire | LS9 7TF |
| Dr Heather Payne | Payne | UNIVERSITY COLLEGE HOSPITAL | 239 Euston Rd, Fitzrovia, London | NW1 2BU |
| Dr David Peake | Peake | CITY HOSPITAL, BIRMINGHAM | Dudley Road, Birmingham | B18 7HQ |
| Dr I Pedley | Pedley | NEWCASTLE GENERAL HOSPITAL | Westgate Road, Newcastle-upon-Tyne | NE4 6BE |
| Dr Clive Peedell | Peedell | THE JAMES COOK UNIVERSITY HOSPITAL | Marton Road, Middlesbrough | TS4 3BW |
| Mr A Pengelly | Pengelly | BATTLE HOSPITAL | Oxford Road, Reading, Berkshire | RG3 1AG |
| Mr Amjad M Peracha | Peracha | ROYAL DERBY HOSPITAL | Derbyshire Royal Infirmary, London Road, Derby | DE1 2QY |
| Dr Matthew Perry | Perry | GUY'S HOSPITAL | St Thomas Street, London | SE1 9RT |
| Mr Raj Persad | Persad | BRISTOL ROYAL INFIRMARY | Marlborough Street, Bristol | BS2 8HW |
| Mr John Peters | Peters | WHIPPS CROSS UNIVERSITY HOSPITAL | Whipps Cross Road, Leytonstone, London | E11 1NR |
| Mr B Pettersson | Pettersson | COUNTESS OF CHESTER HOSPITAL | The Countess Of Chester Health Park , Chester, Cheshire | CH2 1HJ |
| Dr Phan | Phan | WITHYBUSH GENERAL HOSPITAL | Ward 10, Fishguard Road, Haverford West, Pembrokeshire | SA61 2PZ |
| Mr N H Philp | Philp | NEW CROSS HOSPITAL | Wednesfield Road, Wolverhampton, West Midlands | WV10 0QP |
| Mr T Philp | Philp | WHIPPS CROSS UNIVERSITY HOSPITAL | Whipps Cross Road, Leytonstone, London | E11 1NR |
| Mr Jaspal Phull | Phull | ROYAL UNITED HOSPITAL BATH | Combe Park, Bath | BA1 3NG |
| Dr Lisa M Pickering | Pickering | ST GEORGE'S HOSPITAL | Blackshaw Road, Tooting, London | SW17 0RE |
| Dr Katharine Pigott | Pigott | ROYAL FREE HOSPITAL | Pond Street, London | NW3 2QG |
| Dr Elias Pintus | Pintus | WEXHAM PARK HOSPITAL | Wexham street, Slough, Berkshire | SL2 4HL |
| Mr R Plail | Plail | CONQUEST HOSPITAL | The Ridge, St Leonards on Sea, Hastings, East Sussex | TN37 7RD |
| Dr Georgios Plataniotis | Plataniotis | QUEEN'S HOSPITAL | Rom Valley Way, Romford, Essex | RM7 0AG |
| Dr P Nicholas Plowman | Plowman | ST BARTHOLOMEWS HOSPITAL | West Smithfield, London | EC1A 7BE |
| Mr Richard D Pocock | Pocock | ROYAL DEVON & EXETER HOSPITAL (WONFORD) | Barrack Rd, Exeter, Devon | EX2 5DW |
| Mr A J Pope | Pope | HILLINGDON HOSPITAL | Pield Heath Road, Uxbridge | UB8 3NN |
| Mr Rick Popert | Popert | GUY'S HOSPITAL | St Thomas Street, London | SE1 9RT |
| Dr Emilio Porfiri | Porfiri | CITY HOSPITAL | Dudley Road, Birmingham | B18 7HQ |
| Mr Tim Porter | Porter | YEOVIL DISTRICT HOSPITAL | Higher Kingston, Yeovil, Somerset | BA21 4AT |
| Mr John M Potter | Potter | NORTHAMPTON GENERAL HOSPITAL | Billing Rd, Northampton | NN1 5BD |
| Mr Christopher Powell | Powell | COUNTESS OF CHESTER HOSPITAL | The Countess Of Chester Health Park , Chester, Cheshire | CH2 1HJ |
| Dr Thomas B Powles | Powles | WHIPPS CROSS UNIVERSITY HOSPITAL | Whipps Cross Road, Leytonstone, London | E11 1NR |
| Mr Seshadri Sri Prasad | Prasad | DARENT VALLEY HOSPITAL | Darenth Wood Road, Darford, Kent | DA2 8DA |

| | | | | |
|---|---|---|---|---|
| Mr Krishna Prasad | Prasad | GEORGE ELLIOTT HOSPITAL | College Street, Nuneaton, Warks | CV10 7BL |
| Dr R Prashant | Prashant | ROYAL SHREWSBURY HOSPITAL | Mytton Oak Road, Shrewsbury | SY3 8XQ |
| Mr J W Prejbisz | Prejbisz | SOUTHEND UNIVERSITY HOSPITAL | Prittlewell Chase, Westcliff on Sea, Essex | SS0 0RY |
| Mr Stephen Prescott | Prescott | ST JAMES'S UNIVERSITY HOSPITAL | Beckett Street, Leeds, West Yorkshire | LS9 7TF |
| Dr Andrew Protheroe | Protheroe | CHURCHILL HOSPITAL | Old Road, Headington, Oxford | OX3 7LJ |
| Mr Tas Qureshi | Quereshi | QUEEN ALEXANDRA HOSPITAL | Southwick Hill Road, Cosham, Porthmouth, Hampshire | PO6 3LY |
| Mr Khaver N Qureshi | Qureshi | GARTNAVEL GENERAL HOSPITAL | 1053 Great Western Road, Glasgow | G12 0YN |
| Dr Nigel Raby | Raby | ROYAL DEVON & EXETER HOSPITAL (WONFORD) | Barrack Rd, Exeter, Devon | EX2 5DW |
| Dr Narasimhan Ragavan | Ragavan | ROYAL PRESTON HOSPITAL | Sharoe Green Lane North, Fulwood, Preston, Lancashire | PR2 9HT |
| Mr Paul H **Rajjayabun** | Rajjayabun | KIDDERMINSTER HOSPITAL | Bewdley Road , Kidderminster, Worcestershire | DY11 6RJ |
| Mr Palaniappa G S Raju | Raju | CROYDON UNIVERSITY HOSPITAL | Research Office, 1st Floor, Woodcroft Wing, Croydon University Hospital, Croydon | CR7 7YE |
| Dr Prakash B Ramachandra | Ramachandra | RUSSELLS HALL HOSPITAL | Pensnett Road, Dudley, West Midlands | DY1 2HQ |
| Mr R Ramachandaran | Ramachandaran | BASILDON UNIVERSITY HOSPITAL | Nethermayne, Basildon, Essex | SS16 5NL |
| Dr Ramamoorthy | Ramamoorthy | WESTMORLAND GENERAL HOSPITAL | Burton Rd , Kendal | LA9 7RG |
| Dr R Raman | Raman | KENT AND CANTERBURY HOSPITAL | Ethelbert Road, Canterbury, Kent | CT1 3NG |
| Mr Vijay Ramani | Ramani | WITHINGTON HOSPITAL | Nell Lane, Whitington, Manchester | M20 2LR |
| Mr Ramkumar | Ramkumar | ROYAL BOLTON HOSPITAL | Minerva Road, Farnworth, Bolton, Lancs | BL4 0JR |
| Dr A Ramsden | Ramsden | DERRIFORD HOSPITAL | Derriford Road, Plymouth | PL6 8DH |
| Mr Abhay Rane | Rane | EAST SURREY HOSPITAL | Canada Ave, Redhill, Surrey | RH1 5RH |
| Dr Julia Rankin | Rankin | ROYAL DEVON & EXETER HOSPITAL (WONFORD) | Barrack Rd, Exeter, Devon | EX2 5DW |
| Mr Y Rao | Rao | STAFFORDSHIRE GENERAL HOSPITAL | Weston Road, Stafford, Staffordshire | ST16 3SA |
| Mr Hari L Ratan | Ratan | ROYAL DERBY HOSPITAL | Derbyshire Royal Infirmary, London Road, Derby | DE1 2QY |
| Mr Ramachandran Ravi | Ravi | BASILDON & THURROCK UNIVERSITY HOSPITALS NHS TRUST | Nethermayne, Basildon, Essex | SS16 5NL |
| Dr K Ravishankar | Ravishankar | DONCASTER ROYAL INFIRMARY | Doncaster Royal Infirmary, C Block, Doncaster Royal Infirmary, Armthorpe Road, Doncaster | DN2 5LT |
| Dr Read | Read | ROYAL PRESTON HOSPITAL | Sharoe Green Lane North, Fulwood, Preston, Lancashire | PR2 9HT |
| Mr Paul J Reddy | Reddy | MAIDSTONE HOSPITAL | Hermitage Lane, Maidstone, Kent | M16 9QQ |
| Mr Peter R Rimington | Rimington | EASTBOURNE DISTRICT GENERAL HOSPITAL | King's Drive, Eastbourne, East Sussex | BN21 2UD |
| Dr Yvonne Rimmer | Rimmer | WEST SUFFOLK HOSPITAL | Hardwicke Lane, Bury St Edmunds | IP33 2QZ |
| Dr Peter A Ritchie | Ritchie | GLOUCESTERSHIRE ROYAL HOSPITAL | Great Western Road, Gloucester, Gloucestershire | GL1 3NN |
| Dr J Trevor Roberts | Roberts | NEWCASTLE GENERAL HOSPITAL | Westgate Road, Newcastle-upon-Tyne | NE4 6BE |
| Mr Andrew Robertson | Robertson | SCARBOROUGH GENERAL HOSPITAL | Woodlands Drive, Scarborough | YO12 6QL |
| Dr Angus Robinson | Robinson | ROYAL SUSSEX COUNTY HOSPITAL | Eastern Road, Brighton, East Sussex | BN2 5BE |
| Dr Anne C Robinson | Robinson | SOUTHEND UNIVERSITY HOSPITAL | Prittlewell Chase, Westcliff on Sea, Essex | SS0 0RY |
| Mr Lee Q Robinson | Robinson | WARRINGTON HOSPITAL | Lovely Lane, Warrington | WA5 1QG |
| Mr Peter Robson | Robson | UNIVERSITY HOSPITAL AINTREE | Lower Lane, Fazakarley, Liverpool | L9 7AL |
| Mr Mark A Rochester | Rochester | NORFOLK & NORWICH UNIVERSITY HOSPITAL | Colney Lane, Norwich | NR4 7UZ |
| Mr Karol Rogawski | Rogawski | HUDDERSFIELD ROYAL INFIRMARY | Lindley, Huddersfield | HD3 3EA |
| Mr P B Rogers | Rogers | ROYAL BERKSHIRE HOSPITAL | London Road, Reading, Berkshire | RG1 5AN |
| Mr Tomas P Rosenbaum | Rosenbaum | EALING HOSPITAL | Research and Development, Uxbridge Road, Southall, Middlesex | UB1 3HW |
| Dr Nicola Rosenfelder | Rosenfelder | BARNET HOSPITAL | Wellhouse Lane, Barnet, Herts | EN5 3DJ |
| Mr Neil Rothwell | Rothwell | BLACKPOOL VICTORIA HOSPITAL | Whinney Heys Road, Blackpool, Lancashire | FY3 8NR |
| Mr Carl Rowbotham | Rowbotham | ROYAL LANCASTER INFIRMARY | Ashton Road, Lancaster, Lancashire | LA1 4RP |
| Mr Rowe | Rowe | SOUTHMEAD HOSPITAL | Westbury-on-Trym, Bristol, Somerset | BS10 5NB |
| Dr Kathryn Rowley | Rowley | VELINDRE HOSPITAL | Whitchurch, Cardiff, Wales | CF14 2TL |
| Dr Deborah Ruddy | Ruddy | GUY'S HOSPITAL | St Thomas Street, London | SE1 9RT |
| Mr John Rundle | Rundle | ROYAL BOURNEMOUTH HOSPITAL | Castle Lane East, Bournemouth, Dorset | BH7 7DW |
| Dr John M Russell | Russell | BEATSON ONCOLOGY CENTRE | Western Infirmary, Dumbarton Road, Glasgow | G11 6NT |
| Mr P G Ryan | Ryan | CITY HOSPITAL, BIRMINGHAM | Dudley Road, Birmingham | B18 7HQ |
| Dr A Sabharwal | Sabarwal | STOKE MANDEVILLE HOSPITAL | Mandeville Road, Aylesbury, Buckinghamshire | HP21 8AL |
| Dr Sabbagh | Sabbagh | ROYAL SHREWSBURY HOSPITAL | Mytton Oak Road, Shrewsbury | SY3 8XQ |
| Dr A Sabharwal | Sabharwal | CHURCHILL HOSPITAL | Old Road, Headington, Oxford | OX3 7LJ |
| Dr Anand K Saggar | Saggar | ST GEORGE'S HOSPITAL | Blackshaw Road, Tooting, London | SW17 0RE |
| Mr Mohammed El Saghir | Saghir | SALISBURY DISTRICT HOSPITAL | Odstock Road, Salisbury, Wilts | SP2 8BJ |
| M Asad Saleemi | Saleemi | LUTON & DUNSTABLE HOSPITAL | Lewsey Road, Luton | LU4 0DZ |
| Dr Ali Samanci | Samanci | RUSSELLS HALL HOSPITAL | Pensnett Road, Dudley, West Midlands | DY1 2HQ |
| Dr D Sanders | Sanders | KING'S MILL HOSPITAL | Mansfield Road , Sutton-In-Ashfield, Nottinghamshire, | NG17 4JL |
| Mr Sarbjinder Sandhu | Sandhu | KINGSTON HOSPITAL | Galsworthy Road, Kingston-upon-Thames, Surrey | KT2 7QB |
| Mr Vijay K Sangar | Sangar | WITHINGTON HOSPITAL | Southmoor Road,Wythenshawe,Manchester | M23 9LT |
| Mr B Dev Sarmah | Sarmah | GOOD HOPE HOSPITAL | Rectory Road, Sutton Coldfield, West Midlands | B75 7RR |
| Dr D Saunders | Saunders | KING'S MILL HOSPITAL | Mansfield Road , Sutton-In-Ashfield, Nottinghamshire, | NG17 4JL |
| Dr Saw | Saw | WITHYBUSH GENERAL HOSPITAL | Ward 10, Fishguard Road, Haverford West, Pembrokeshire | SA61 2PZ |

| | | | | |
|---|---|---|---|---|
| Mr M F Saxby | Saxby | CITY GENERAL HOSPITAL, STOKE ON TRENT | Newcastle Road, Stoke-on-Trent | ST4 6QG |
| Dr Sayers | Sayers | COUNTY HOSPITAL | Egerton Rd, Guildford | GU2 7XX |
| Mr Hartwig Schwaibold | Schwaibold | SOUTHMEAD HOSPITAL | Westbury-on-Trym, Bristol, Somerset | BS10 5NB |
| Dr John E Scoble | Scoble | STAFFORDSHIRE GENERAL HOSPITAL | Weston Road, Stafford, Staffordshire | ST16 3SA |
| Dr Christopher Scrase | Scrase | IPSWICH HOSPITAL | Heath Road, Ipswich, Suffolk | IP4 5PD |
| Mr C Seipp | Seipp | WREXHAM MAELOR HOSPITAL | Croesnewydd Road, Wrexham | LL13 7TD |
| Mr Selim | Selim | MILTON KEYNES GENERAL HOSPITAL | Standing  Way, Eaglestone, Milton Keynes | MK6 5LD |
| Mr Henry Sells | Sells | DERRIFORD HOSPITAL | Derriford Road, Plymouth | PL6 8DH |
| Dr Semkind | Semkind | CHELTENHAM GENERAL HOSPITAL | Sandford Road, Cheltenham, Gloucester | GL53 7AG |
| Mr Krishna K Sethia | Sethia | NORFOLK & NORWICH UNIVERSITY HOSPITAL | Colney Lane, Norwich | NR4 7UZ |
| Mr David C Shackley | Shackley | SALFORD ROYAL HOSPITAL NHS FOUNDATION TRUST | Stott Lane, Salford | M6 1XX |
| Dr Shaffer | Shaffer | BASINGSTOKE AND NORTH HAMPSHIRE HOSPITAL | Aldermaston Road, Basingstoke, | RG24 9NA |
| Dr Shafii | Shafii | WITHYBUSH GENERAL HOSPITAL | Charles Hastings Way, Worcs | WR5 1DD |
| Dr Nihil Shah | Shah | HEMEL HEMPSTEAD GENERAL HOSPITAL | Hillfield Road, Hemel Hempstead, Herts | HP2 4AD |
| Dr D Shakespeare | Shakespeare | THE JAMES COOK UNIVERSITY HOSPITAL | South Tees NHS Foundation Trust, Dept Radiotherapy & Oncology | TS4 3BW |
| Dr Sue Shanley | Shanley | ROYAL MARSDEN HOSPITAL | Fulham Road, London | SW3 6JJ |
| Mr Neerah K Sharma | Sharma | ROYAL OLDHAM HOSPITAL | Rochdale Road, Oldham, Greater Manchester | OL1 2JH |
| Dr Denise J Sheehan | Sheehan | NORTH DEVON DISTRICT HOSPITAL | Raleigh Park, Barnstaple, Devon | EX31 4JB |
| Mr I Shergill | Shergill | WREXHAM MAELOR HOSPITAL | Croesnewydd Road, Wrexham | LL13 7TD |
| Mr Matin Sheriff | Sheriff | MEDWAY MARITIME HOSPITAL | Windmill Road, Gillingham | ME7 5NY |
| Dr Elizabeth Sherwin | Sherwin | WEST SUFFOLK HOSPITAL | Hardwick Lane, Bury St Edmunds | IP33 2QZ |
| Mr Ben Sherwood | Sherwood | NOTTINGHAM UNIVERSITY HOSPITAL NHS TRUST- CITY CAMPUS | Hucknall Road, Nottingham | NG5 1PB |
| Mr David Shiritone | Shiritone | CHESTERFIELD ROYAL HOSPITAL | Calow, Chesterfield, Derbyshire | S44 5BL |
| Dr Balasubramanian T Srinivasan | Shrinivasan | LINCOLN COUNTY HOSPITAL | Greetwell Road, Lincoln, Lincolnshire | LN2 5QY |
| Dr Poh Lin Shum | Shum | BELFAST CITY HOSPITAL | Lisburn Road, Belfast, Northern Ireland | BT9 7AB |
| Dr LucySide | Side | GREAT ORMOND STREET HOSPITAL | Great Ormond Street Hospital, Great Ormond Street | WC1N 3JH |
| Dr Norma Sidek | Sidek | CUMBERLAND INFIRMARY | Cumberland Infirmary, Newtown Road, Carlisle, Cumbria | CA2 7HY |
| Professor Karol Sikora | Sikora | HAMMERSMITH HOSPITAL | Du Cane Road, London | W12 0HS |
| Dr R Simcock | Simcock | ST THOMAS'S HOSPITAL | Lambeth Palace Road, London | SE1 7EH |
| Mr Matthew Simms | Simms | CASTLE HILL HOSPITAL | Castle Road, Cottingham, East Yorkshire | HU16 5JQ |
| Mr Andrew M Sinclair | Sinclair | STEPPING HILL HOSPITAL | Poplar Grove, Hazel Grove, Stockport | SK2 7JE |
| Mr Pravin Singh | Singh | HARROGATE DISTRICT HOSPITAL | Lancaster Park Road, Harrogate | HG2 7SX |
| Dr M Siva | Siva | BEATSON ONCOLOGY CENTRE | Western Infirmary, Dumbarton Road, Glasgow | G11 6NT |
| Dr Bruce Sizer | Sizer | COLCHESTER GENERAL HOSPITAL | Turner Road, Colchester, Essex | CO4 5JL |
| Ms D Skennerton | Skennerton | ST HELIER HOSPITAL | Wrythe Green Lane,Carshalton, Surrey | SM5 1AA |
| Dr Robert Skyrme | Skyrme | PRINCESS OF WALES HOSPITAL | Coity Road, Bridgend | CF31 1RQ |
| Mr Michael F Smith | Smith | FALKIRK & DISTRICT ROYAL INFIRMARY | Major's Loan, Falkirk, Scotland | FK1 5QE |
| Mr James Smith | Smith | MATER HOSPITAL | 47-51 Crumlin Road, Belfast | BT14 6AB |
| Dr Michael Sokal | Sokal | NOTTINGHAM CITY HOSPITAL | Hucknall Road, Nottingham | NG5 1PB |
| Mr Graham M Sole | Sole | HEREFORD COUNTY HOSPITAL | Union Walk, Hereford | HR1 2ER |
| Mr L Z Solomon | Solomon | ST MARY'S HOSPITAL (ISLE OF WIGHT) | Parkhurst Road , Newport, Isle of Wight | PO30 5TG |
| Mr Naeem Soomro | Soomro | WEST CUMBERLAND HOSPITAL | Homewood, Hensingham, Whitehaven | CA28 8JG |
| Mr Mark J Speakman | Speakman | TAUNTON AND SOMERSET HOSPITAL | Musgrove Park, Taunton, Somerset | TA1  5DA |
| Dr Alexander Spiers | Spiers | ROYAL DEVON & EXETER HOSPITAL (WONFORD) | Barrack Rd, Exeter, Devon | EX2 5DW |
| Dr Thiagarajan Sreenivasan | Sreenivasan | LINCOLN COUNTY HOSPITAL | Cliff Gardens, Scunthorpe, N Lincolnshire | DN15 7BH |
| Dr N Srihari | Srihari | ROYAL SHREWSBURY HOSPITAL | Mytton Oak Road, Shrewsbury | SY3 8XQ |
| Dr Narayanan N Srihari | Srihari | ROYAL SHREWSBURY HOSPITAL | Mytton Oak Road, Shrewsbury | SY3 8XQ |
| Mr Srinivasan | Srinivasan | GLAN CLWYD HOSPITAL | Bodelwyddan, Rhyl, Wales | LL18 5UJ |
| Professor Seshadri Sriprasad | Sriprasad | DARENT VALLEY HOSPITAL | Darenth Wood Road, Dartford, Kent | DA2 8DA |
| Mr Rajagopalan Sriram | Sriram | UNIVERSITY HOSPITAL OF COVENTRY AND WARWICKSHIRE | Clifford Bridge Road, Walsgrave, Coventry | CV2 2DX |
| Dr John N Staffurth | Staffurth | VELINDRE HOSPITAL | Whitchurch, Cardiff, Wales | CF14 2TL |
| Mr Richard Stephenson | Stephenson | ARROWE PARK HOSPITAL | Arrowe Park Road, Upton , Wirral, Merseyside | CH49 5PE |
| Dr D Stewart | Stewart | BELFAST CITY HOSPITAL | Lisburn Road, Belfast, Northern Ireland | BT9 7AB |
| Dr Andrew Stockdale | Stockdale | UNIVERSITY HOSPITAL OF COVENTRY AND WARWICKSHIRE | Clifford Bridge Road, Walsgrave, Coventry | CV2 2DX |
| Mr Mark A Stott | Stott | ROYAL DEVON & EXETER HOSPITAL (WONFORD) | Barrack Rd, Exeter, Devon | EX2 5DW |
| Mr M J Stower | Stower | YORK DISTRICT HOSPITAL | Wiggington Road, York, Yorkshire | YO31 8HE |
| Mr John R Strachan | Strachan | WARWICK HOSPITAL | Lakin Road, Warwick, Warks | CV34 5BW |
| Mr Edward Streeter | Streeter | KENT& CANTERBURY HOSPITAL | Ethelbert Road, Canterbury, Kent | CT1 3NG |
| Professor Nicholas S A Stuart | Stuart | YSBYTY GWYNEDD | Penrhosgarnedd, Bangor | LL57 2PW |
| Dr Elaine Sugden | Sugden | CHURCHILL HOSPITAL | Old Road, Headington, Oxford | OX3 7LJ |
| Dr Yae-Eun Suh | Suh | KINGSTON HOSPITAL | Galsworthy RoadKingston upon Thames, Surrey | KT2 7QB |

| | | | | |
|---|---|---|---|---|
| Mr Duncan Summerton | Summerton | LEICESTER GENERAL HOSPITAL | Gwendolen Road, Leicester | LE5 4PW |
| Dr Santhanam Sundar | Sundar | NOTTINGHAM CITY HOSPITAL | Hucknall Road, Nottingham | NG5 1PB |
| Mr S K Sundaram | Sundaram | PINDERFIELDS HOSPITAL | Aberford Road, Wakefield, West Yorkshire | WF1 4DG |
| Mr Gokarakonda Suresh | Suresh | JAMES PAGET HOSPITAL | Lowestoft Road, Gorleston, Great Yarmouth, Norfolk | NR31 6LA |
| Dr Surrange | Surrange | ROYAL OLDHAM HOSPITAL | Rochdale Road, Oldham, Greater Manchester | OL1 2JH |
| Mr Shabbir Susnerwala | Susnerwala | BLACKPOOL VICTORIA HOSPITAL | Whinney Heys Road, Blackpool, Lancashire | FY3 8NR |
| Mr Kuchibhotla S Swami | Swami | ABERDEEN ROYAL INFIRMARY | Foresterhill, Aberdeen | AB25 22N |
| Mr Michael Swinn | Swinn | EAST SURREY HOSPITAL | Canada Ave, Redhill, Surrey | RH1 5RH |
| Mr Haider Syed | Syed | HEARTLANDS HOSPITAL | Bordesley Green East, Birmingham | B9 5SS |
| Miss Stephanie J Symons | Symons | PINDERFIELDS HOSPITAL | Aberford Road, Wakefield, West Yorkshire | WF1 4DG |
| Dr Isabel Syndikus | Syndikus | CLATTERBRIDGE CENTRE FOR ONCOLOGY | Clatterbridge Rd,  Bebington, Wirral | CH63 4JY |
| Dr Saad Tahir | Tahir | BROOMFIELD HOSPITAL | Court Road, Broomfield, Chelmsford, Essex | CM1 5ET |
| Mr Chandran Tanabalan | Tanabalan | HOMERTON UNIVERSITY HOSPITAL | Homerton Row, London | E9 6SR |
| Mr Sanjeev Taneja | Taneja | LUTON & DUNSTABLE HOSPITAL | Lewsey Road, Luton | LU4 0DZ |
| Dr J Tanquay | Tanquay | VELINDRE HOSPITAL | Whitchurch, Cardiff, Wales | CF14 2TL |
| Dr Katherine Tarver | Tarver | QUEEN'S HOSPITAL | Rom Valley Way, Romford, Essex | RM7 0AG |
| Dr John W Taylor | Taylor | ST GEORGE'S HOSPITAL | Blackshaw Road, Tooting, London | SW17 0QT |
| Dr J W Taylor | Taylor | WESTERN GENERAL HOSPTIAL | Crewe Road, Edinburgh | EH4 2UX |
| Mr Seamus Teahan | Teahan | FORTH VALLEY ROYAL HOSPITAL | Stirling Road, Larbert | FK5 4WR |
| Mr T Terry | Terry | LEICESTER GENERAL HOSPITAL | Gwendolen Road, Leicester | LE5 4PW |
| Mr Mohanarangam Thangavelu | Thankgavelu | YSBYTY GWYNEDD | Ysbyty Gwynedd, Bangor, Gwynedd | LL57 2PW |
| Dr Robert J Thomas | Thomas | BEDFORD HOSPITAL | Primrose Oncology Unit, Kempston Road, Bedford | MK42 9DJ |
| Mr Stephen A Thomas | Thomas | DERBYSHIRE ROYAL INFIRMARY | London Road, Derby | DE1 2QY |
| Mr Alan Thompson | Thompson | KINGSTON HOSPITAL | Galsworthy Road, Kingston-upon-Thames, Surrey | KT2 7QB |
| Dr Alastair H Thomson | Thomson | ROYAL CORNWALL HOSPITAL | Treliske, Truro, Cornwall | TR1 3LJ |
| Dr A Thurston | Thurston | ST ALBANS CITY HOSPITAL | Waverley Road, St Albans | AL3 5PN |
| Dr Owen Tilsley | Tilsley | VELINDRE HOSPITAL | Whitchurch, Cardiff, Wales | CF14 2TL |
| Mr Stuart  F Tindall | Tindall | DIANA PRINCESS OF WALES HOSPITAL | Scartho Road, Grimsby | DN33 2BA |
| Dr K Tipples | Tipples | ST BARTHOLOMEW'S HOSPITAL | West Smithfield, London | EC1A 7BE |
| Dr Marc Tischokowitz | Tischkowitz | ADDENBROOKE'S HOSPITAL | Cambridge Biomedical Campus, Hill Road, Cambridge | CB2 0QQ |
| Dr Shaun Tolan | Tolan | THE CLATTERBRIDGE CANCER CENTRE | Clatterbridge Rd,  Bebington, Wirral | CH63 4JY |
| Dr Tong | Tong | GUY'S HOSPITAL | St Thomas Street, London | SE1 9RT |
| Mr Hamid Toussi | Toussi | GLAN CLWYD HOSPITAL | Bodelwyddan, Rhyl, Wales | LL18 5UJ |
| Dr Elizabeth W Toy | Toy | NORTH DEVON DISTRICT HOSPITAL | Raleigh Park, Barnstaple, Devon | EX31 4JB |
| Dr Sayers | Tran | COUNTY HOSPITAL | Egerton Rd, Guildford | GU2 7XX |
| Dr Sarah Treece | Treece | PETERBOROUGH CITY HOSPITAL | Edith Cavell Campus, Bretton Gate, Peterborough | PE3 9GZ |
| Professor Richard C Trembath | Trembath | LEICESTER ROYAL INFIRMARY | Infirmary Square, Leicester | LE1 5WW |
| Dr David Tsang | Tsang | BASILDON UNIVERSITY HOSPITAL | Nethermayne, Basildon, Essex | SS16 5NL |
| Mr David N Tulloch | Tulloch | WESTERN GENERAL HOSPTIAL | Crewe Road, Edinburgh | EH4 2UX |
| Mr Kevin J Turner | Turner | ROYAL BOURNEMOUTH HOSPITAL | Castle Lane East, Bournemouth, Dorset | BH7 7DW |
| Mr James Tweedle | Tweedle | FALKIRK & DISTRICT ROYAL INFIRMARY | Major's Loan, Falkirk, Scotland | FK1 5QE |
| Dr C J Tyrell | Tyrell | DERRIFORD HOSPITAL | Derriford Road, Plymouth | PL6 8DH |
| Mr N Umez-Eronini | Umez-Eronini | CUMBERLAND INFIRMARY | Cumberland Infirmary, Newtown Road, Carlisle, Cumbria | CA2 7HY |
| Mr Graeme H Urwin | Urwin | NUFFIELD HOSPITAL YORK | Haxby Road, York | YO31 8TA |
| Mr Justin A Vale | Vale | ST MARY'S HOSPITAL, LONDON | Praed Street, Paddington, London | W2 1NY |
| Dr Van As | Van | KINGSTON HOSPITAL | Galsworthy Road, Kingston-upon-Thames, Surrey | KT2 7QB |
| Dr Nicholas Van As | Van As | KINGSTON HOSPITAL | Galsworthy Road, Kingston-upon-Thames, Surrey | KT2 7QB |
| Dr Mohini Varughese | Varughese | MUSGROVE PARK HOSPITAL | Mugrove Park, Taunton | TA1 5DA |
| Dr Subramaniam Vasanthan | Vasanthan | LEICESTER GENERAL HOSPITAL | Gwendolen Road, Leicester | LE5 4PW |
| Dr Ramchandran Venkitaraman | Venkitaraman | THE IPSWICH HOSPITAL NHS TRUST | Heath Road, Ipswich, Suffolk | IP4 5PD |
| Dr Balaji Venugopal | Venugopal | BEATSON WEST OF SCOTLAND CANCER CENTRE | Western Infirmary, Dumbarton Road, Glasgow | G11 6NT |
| Mr Sean Vesey | Vesey | SOUTHPORT & FORMBY DGH | Town Lane, Southport | PR8 6PN |
| Dr Maria Vilarino-Varela | Vilarino-Varela | ROYAL FREE HOSPITAL | Pond Street, London | NW3 2QG |
| Dr John Violet | Violet | THE GREAT WESTERN HOSPITAL | Marlborough Road, Swindon, Wilts | SN3 6BB |
| Mr Jaspal Virdi | Virdi | CAPIO RIVERS HOSPITAL | High Wych Road, Sawbridgeworth | CM21 0HH |
| Dr Anil Vohra | Vohra | BASILDON UNIVERSITY HOSPITAL | Nethermayne, Basildon, Essex | SS16 5NL |
| Dr Robert Wade | Wade | NORFOLK & NORWICH UNIVERSITY HOSPITAL | Colney Lane, Norwich | NR4 7UZ |
| Miss Elizabeth Waine | Waine | ROYAL DEVON & EXETER HOSPITAL (WONFORD) | Barrack Rd, Exeter, Devon | EX2 5DW |
| Dr Katherine Waite | Waite | ADDENBROOKE'S HOSPITAL | Hills Road, Cambridge | CB2 2QQ |
| Mr E M Walker | Walker | MILTON KEYNES GENERAL HOSPITAL | Standing Way, Eaglestone, Milton Keynes | MK6 5LD |
| Mr Roger Walker | Walker | ST HELIER HOSPITAL | Wrythe Green Lane,Carshalton, Surrey | SM5 1AA |

| | | | | |
|---|---|---|---|---|
| Mr David M A Wallace | Wallace | QUEEN ELIZABETH HOSPITAL | Mindelsohn Way, Edgbaston, Birmingham | B15 2TH |
| Mr Tom Walton | Walton | NOTTINGHAM UNIVERSITY HOSPITAL NHS TRUST- CITY CAMPUS | Hucknall Road, Nottingham | NG5 1PB |
| Ms Hazel Warburton | Warburton | WITHINGTON HOSPITAL | Nell Lane, Whitington, Manchester | M20 2LR |
| Mr Nicholas A Watkin | Watkin | ST ANTHONY'S HOSPITAL | London Road, North Cheam | SM3 9DW |
| Mr M E Watson | Watson | ROYAL PRESTON HOSPITAL | Sharoe Green Lane North, Fulwood, Preston, Lancashire | PR2 9HT |
| Professor J H Waxman | Waxman | HAMMERSMITH HOSPITAL | Du Cane Road, London | W12 0HS |
| Mr Brian Waymont | Waymont | NEW CROSS HOSPITAL | Wednesfield Road, Wolverhampton, West Midlands | WV10 0QP |
| Dr Andrew Weaver | Weaver | WYCOMBE GENERAL HOSPITAL | Queen Alexander Rd, High Wycombe, Buckinghamshire | HP11 2TT |
| Mr Ralph J Webb | Webb | NORFOLK & NORWICH UNIVERSITY HOSPITAL | Colney Lane, Norwich | NR4 7UZ |
| Mrs Robyn Webber | Webber | QUEEN MARGARET HOSPITAL | Whitefield Road, Dunfermline | KY12 0SU |
| Mr John Webster | Webster | NORTHWICK PARK HOSPITAL | Watford Road, Harrow | HA1 3UJ |
| Mr Andrew Wedderburn | Wedderburn | ROYAL BOURNEMOUTH HOSPITAL | Castle Lane East, Bournemouth, Dorset | BH7 7DW |
| Dr Paula Wells | Wells | WHIPPS CROSS UNIVERSITY HOSPITAL | Whipps Cross Road, Leytonstone, London | E11 1NR |
| Mr G D Wemyss-Holden | Wemyss-Holden | BLACKBURN ROYAL INFIRMARY | Haslingden Road, Blackburn | BB2 3HH |
| Dr Charlotte Westbury | Westbury | MOUNT VERNON CANCER CENTRE | Rickmansworth Road, Northwood, Middlesex | HA6 2RN |
| Mr P M T Weston | Weston | PINDERFIELDS HOSPITAL | Aberford Road, Wakefield, West Yorkshire | WF1 4DG |
| Dr Duncan Wheatley | Wheatley | ROYAL CORNWALL HOSPITAL | Treliske, Truro, Cornwall | TR1 3LJ |
| Mr P Whelan | Whelan | ST JAMES' UNIVERSITY HOSPITAL | Beckett Street, Leeds, West Yorkshire | LS9 7TF |
| Dr D Whillis | Whillis | RAIGMORE HOSPITAL | Perth Road, Inverness, Scotland | IV2 3UJ |
| Mr Adam D Wilde | Wilde | ROYAL CORNWALL HOSPITAL | Treliske, Truro, Cornwall | TR1 3LJ |
| Dr Vicki Wiles | Wiles | ADDENBROOKE'S HOSPITAL | Cambridge Biomedical Campus, Hill Road, Cambridge | CB2 0QQ |
| Dr Marie Wilkins | Wilkins | ROYAL SUSSEX COUNTY HOSPITAL | Eastern Road, Brighton, East Sussex | BN2 5BE |
| Mr John H Williams | Williams | DERBY CITY GENERAL HOSPITAL | Uttoxeter Road, Derby | DE22 3NE |
| Mr Simon Williams | Williams | DERBY CITY GENERAL HOSPITAL | Uttoxeter Road, Derby | DE22 3NE |
| Mr Michael Willis | Willis | UNIVERSITY HOSPITAL OF COVENTRY AND WARWICKSHIRE | Clifford Bridge Road, Walsgrave, Coventry | CV2 2DX |
| Mr Michael I Wills | Wills | WALSGRAVE HOSPITAL | Clifford Bridge Road, Walsgrave, Coventry | CV2 2DX |
| Mr Richard Wilson | Wilson | FURNESS GENERAL HOSPITAL | Dalton Lane, Barrow in Furness | LA14 4LF |
| Mr J R Wilson | Wilson | YORK DISTRICT HOSPITAL | Wiggington Road, York, Yorkshire | YO31 8HE |
| Dr Phyllis Windsor | Windsor | NINEWELLS HOSPITAL | Ninewells Avenue, Dundee | DD1 9SY |
| Mr Mathias H Winkler | Winkler | CHARING CROSS HOSPITAL | Fulham Palace Road, London | W6 8RF |
| Dr Marcus Wise | Wise | ROYAL PRESTON HOSPITAL | Sharoe Green Lane North, Fulwood, Preston, Lancashire | PR2 9HT |
| Mr Simon Woodhams | Woodhams | WORTHING HOSPITAL | Lyndhurst Road, Worthing, West Sussex | BN11 2DH |
| Professor C Woodhouse | Woodhouse | ROYAL MARSDEN HOSPITAL | Fulham Road, London | SW3 6JJ |
| Dr Cathryn Woodward | Woodward | WEST SUFFOLK HOSPITAL | Hardwick Lane, Bury St Edmunds | IP33 2QZ |
| Dr Woolf | Woolf | HOMERTON UNIVERSITY HOSPITAL | Homerton Row, London | E9 6SR |
| Mr K A Woolfenden | Woolfenden | ROYAL LIVERPOOL UNIVERSITY HOSPITAL | Prescot Street, Liverpool | L7 8XP |
| Dr Jane Worlding | Worlding | UNIVERSITY HOSPITAL OF COVENTRY AND WARWICKSHIRE | Clifford Bridge Road, Walsgrave, Coventry | CV2 2DX |
| Mr Mark Wright | Wright | BRISTOL ROYAL INFIRMARY | Marlborough Street, Bristol | BS2 8HW |
| Dr James P Wylie | Wylie | CHRISTIE HOSPITAL | Wilmslow Road, Withington, Manchester | M20 4BX |
| Dr WYLIE | Wylie | THE CHRISTIE HOSPITAL | Wilmslow Road, Withington, Manchester | M20 4BX |
| Dr Chris Wynne | Wynne | ROYAL MARSDEN HOSPITAL | Fulham Road, London | SM2 5PT |
| Mr George Yardy | Yardy | THE IPSWICH HOSPITAL NHS TRUST | Heath Road, Ipswich, Suffolk | IP4 5PD |
| Mr Fadey Youssef | Youssef | CASTLE HILL HOSPITAL | Castle Road, Cottingham, East Yorkshire | HU16 5JQ |
| Mr W. Zafar | Zafar | MACCLESFIELD DISTRICT GENERAL HOSPITAL | Victoria Rd, Macclesfield | SK10 3BL |
| Ms Angelika Zang | Zang | BROMLEY HOSPITAL | Cromwell Avenue, Bromley, Kent | BR2 9AJ |
| Dr A Zarkar | Zarkar | QUEEN ELIZABETH HOSPITAL BIRMINGHAM | Mindelsohn Way, Edgbaston, Birmingham | B15 2TH |
