# Supplementary material for: Relationship of self-reported body size and shape with risk for prostate cancer: A UK case-control study
Source: PLoS One. 2020 Sep 17;15(9):e0238928. doi: 10.1371/journal.pone.0238928 (PMC7498010; doi:10.1371/journal.pone.0238928)
Supplement: S2 File — (DOCX) [file pone.0238928.s002.docx]

Section 10 Further details about you

In this section, we would like to know more about your body size and body shape. This includes the changes of your weight or trouser size in the past years. Please give as approximate estimates if you can and

If you are cases participants, please recall your weight and height **before your diagnosis.**

67) Please can you tell me your current weight and height?

My weight is .....………….. Stones/lbs or ………….. Kgs.

My height is .....…………… ft/ins or…………………… cm.

68) Have you changed your weight over the last 5 years?

yes and my weight was ……………. Stones/Ibs/ or ………….Kgs

no (*go to question 69)*

69) What is your collar-size?

|  | inches |
| --- | --- |

70) Please can you tell me your waist and your approximate hip circumference, either in inches or in centimetres? If you cannot remember your waist circumference, can you recall your trouser size (for example size 30)?

|  | **Waist/ Trouser Size** | | **Hip** | |
| --- | --- | --- | --- | --- |
|  | **inch** | **cm** | **inch** | **cm** |
| **In your 20s** |  |  |  |  |
| **In your 30s** |  |  |  |  |
| **In your 40s** |  |  |  |  |
| **During the last 5 years** |  |  |  |  |

Please select the shape you think you were at different ages. *(Please write down the number you think you were).*

71) In your 20s.

72) In your 30s.

73) In your 40s.

74) During the last 5 years

With the answer of your body shape (questions 71 to 74 ), please select one of the descriptions below that suit you the most at different ages *(please write down number in the box)*

1. ***Apple shape-*** where your body fat is distributed mainly around your tummy area.

***2. Pear shape***- where your body fat is distributed mainly on your hip and thigh.

1. ***Oval shape*-** where your body is distributed around your neck, your chest, your tummy area and also your thigh.

***4. Symmetric shape****-* where you are lean with no fat distribution around your body.

75) My body shape is
